# Abstracts from the 3rd Annual A-CURE Symposium: Chicago, IL, USA. 8-9 November 2018

**DOI:** 10.1186/s12872-018-0978-y

**Published:** 2019-01-28

## A-1 Higher Mortality in Cardiogenic Shock Patients Transferred from a Referring Facility Compared to Patients Presenting to an Academic Medical Center

### Mark Kaeppler**,** Eks W. Pollock, Kadam Patel, Mary Conti, Sergey Tarima, Mitchell Saltzberg, Soo Kim, Lucian Durham, David L. Joyce, Asim Mohammed

#### Medical College of Wisconsin, Milwaukee, WI, USA

##### **Correspondence:** Mark Kaeppler

**Background:** Cardiogenic shock (CS) is a highly fatal condition characterized by cardiac dysfunction leading to inadequate tissue perfusion. Few studies have sought to clarify the rate of mortality among different patient populations hospitalized with CS, and none have investigated CS patients transferred for a higher level of care.

**Hypothesis:** We hypothesized that transfer patients would experience a higher mortality rate compared to inpatients initially admitted to an academic medical center.

**Methods:** Studied patients were hospitalized at an academic medical center with an ICD-9/10 discharge diagnosis of CS between 12/2015 and 8/2017. A chart review identified admission status: to the academic medical center or transferred from a referring facility. Mortality was defined as death in the hospital or as a discharge to hospice. Patient characteristics stratified by admission status were compared. Univariate and multivariate logistic regression analysis were performed. All variables reported as significant had a two-sided p-value ≤0.01, unless otherwise stated.

**Results:** 508 patients were included in this analysis: 62% were male, 73% were Caucasian and mean age was 63 years. Presentation with STEMI was seen in 13% and ACS in 25% of patients. 31% of patients were transferred from another facility. Transfer patients had a higher rate of mortality than non-transfer patients (43.6% vs 33.5%, p=0.03). Univariate predictors of mortality included admission post cardiac arrest, respiratory failure, acute renal failure, need for dialysis, lower SBP or MAP and elevated direct bilirubin, lactic acid or creatinine. Multivariate regression analysis identified admission post cardiac arrest, respiratory failure, acute renal failure, need for dialysis and elevated lactic acid as predictors of mortality. Transfer status was not an independent predictor when adjusted for comorbidities.

**Conclusion:** In the study population, transfer patients have a higher severity of illness. This explains the increased mortality observed in this group, and indicates the need for early aggressive therapy in this population.

## A-2 Prolonged Impella 5.0 support is Safe and Used as a Bridge to Clinical Decision Making

### Daniel Nelson, Asim Mohammed, David Joyce

#### Medical College of Wisconsin, Milwaukee, WI, USA

##### **Correspondence:** Daniel Nelson

**Background:** Mortality from cardiogenic shock remains a significant clinical challenge. Short term mechanical circulatory support devices have potential to improve outcomes. The Impella 5.0 is currently FDA approved for up to 10 days. We report our institutional experience with the Impella 5.0 to improve survival and expand treatment options in patients presenting with severely decompensated end stage heart failure (HF) and cardiogenic shock.

**Hypothesis:** The Impella 5.0 can serve as an effective bridge to recovery or as an optimization tool for long-term support in patients with cardiogenic shock.

**Methods:** A retrospective review was performed on all consecutive patients supported with Impella 5.0 from August 2017 to May 2018 at Froedtert and the Medical College of Wisconsin.

**Results:** A total of 23 Impella 5.0 devices were implanted in men and women as a “bridge to decision” regarding the potential for recovery vs. evaluation for long-term assist device. Thirteen patients presented in cardiogenic shock and 10 in severely decompensated HF. All implantations were performed via axillary approach. The average number of pressors was 0.9 (range 0-3 pressors). The average norepinephrine dose was 0.15 mcg (range 0.04 – 0.33 mcg) and epinephrine 0.10 mcg (range 0.06 - 0.15 mcg). After Impella implantation, patients were on pressors for an average of 1.4 days (range 0 – 4 days). The average length of Impella duration was 14 days (range 0 – 36 days). There were no major adverse complications from prolonged Impella support. No devices malfunctioned and there were no reported strokes or major bleeding events. Three (13%) patients had hemolysis requiring device removal on days 6 and 18 respectively. Thrombocytopenia requiring more than 10 units of platelets occurred in 2 patients (11%). Eight patients (34%) died and fifteen (66%) survived. Of those that survived eight (53%) regained their ejection fraction (EF) and six (26%) had a ventricular assist device (VAD) placed. Of the eight (37%) that died, four were not VAD candidates for psychosocial reasons so decided on hospice/withdrawal of care and other three died of worsening HF and one died of chronic abdominal aortic aneurysm rupture.

**Conclusion:** Prolonged hemodynamic support with Impella 5.0 is a safe and viable option for patients presenting with decompensated HF and cardiogenic shock as a bridge to decision strategy. This type of strategy allows for through evaluation for viable VAD candidates and offers.

## A-3 Use of Impella CP device, a microaxial left ventricular assist device, has been approved for cardiogenic shock. We aim to evaluate feasibility and short-term results of this device in a community hospital setting

### Murad Abdelsalam, Nishtha Sareen, Michele Degregorio, Kiritkumar Patel

#### St. Joseph Mercy Oakland, Pontiac, MI, USA

##### **Correspondence:** Murad Abdelsalam

**Background:** Cardiogenic shock secondary to acute myocardial infarction is associated with high mortality rates. Use of Impella CP device, a microaxial left ventricular assist device, has been approved for cardiogenic shock. We aim to evaluate feasibility and short-term results of this device in a community hospital setting.

**Methods:** A retrospective chart review of 18 patients who underwent left ventricle unloading with the Impella CP device through femoral artery for cardiogenic shock secondary to acute myocardial infarction was performed from June 1, 2016 to June 1 2017. Data were evaluated with regard to baseline and procedural characteristics and also included an assessment of the short term (30 day) and intermediate (1 year) clinical outcomes.

**Results:** A total of 18 patients (13 men), mean age (66.7 +\- 14.16 years) with AMICS. Majority of patients had STEMI (83%), and more than half with cardiac arrest (56%). All patients received Impella device during the initial coronary intervention. Pre-PCI placement was performed in 9 patients (50%), 6 patients (33%) had intra-procedural placement and the reminder (17%) had Post -PCI placement. For patients with STEMI presentation, door to balloon and door to unload times were 76 mins and 74 mins respectively. Shock onset to device time for all patients was 64 mins. The infarct related artery was LAD in 43%, RCA in 28%, LCx in 19% and LM in 10% of patients. Average Cardiac power output (CPO) prior to procedure was 0.35 watts and average Pulmonary artery plasticity index (PAPI) was 0.72. The mean duration of Impella was 49 hours and average ICU stay was 5 days. One patient was transferred for ECMO support, one patient had ischemic stroke. Another patient had hemolysis requiring device explanation, and two patients had limb ischemia requiring external femoral bypass during the same intervention. 30 day survival rate was 61%. Mortality at 12 months was 50%.

**Conclusion:** Left ventricular unloading with the use of Impella CP device in Acute myocardial infarction complicated by cardiogenic shock is feasible and provides beneficial hemodynamic support. Mortality at one year was better than historic data in these critically ill patients.

## A-4 First in Man Implantations of a Newly Designed Transaortic Axial Flow Ventricular Assist Device (Impella 5.5)

### Alexander M. Bernhardt, Samer Hakmi, Christoph Sinning, Edith Lubos, Hermann Reichenspurner

#### University Heart Center, Hamburg, Germany

##### **Correspondence:** Alexander M. Bernhardt

**Background:** The transaortic axial flow device Impella 5.0 (Abiomed, Danvar, Massachussets, USA) is an established and approved short-term device for patients with left ventricular failure in need of mechanical circulatory support, especially in those patients who are in need of full left ventricular circulatory support. However, it is approved for up to seven days only. In certain situations longer duration of support is needed. Therefore, the Impella 5.5 was developed and has currently an approval for 30days of support

**Methods:** Recently, the Impella 5.5 received CE mark approval. The newly designed pump has new motor bearings, new optical flow and pressure sensing and measurement, a shorter and stiffer pump and is lacking a pig-tail catheter at the tip of the pump. Compared to the former Impella 5.0 the only access site for implantation of the Impella 5.5 is the axillary artery.

**Results:** We here present the experience of the first-in-man implantations of the Impella 5.5. So far, a total number of six pumps have been performed worldwide of those the first four in our institution. The updated numbers and results, as well as surgical implant techniques and images, will be presented.

**Conclusion:** The newly designed Impella 5.5 provides full support with a flow up to 5.7 l/min and is approved for up to 30 days. Implantation and placement of the device is feasible and safe. During support, no pump related adverse events occurred. More implantations are planned in Europe to evaluate the encouraging first experiences.

## A-5 Relation of mitral regurgitation severity to thromboembolic risk in atrial fibrillation patients

### Abdelrahman Metwally Sr., Saad Elzoghby, Kamal Merghany, Yasser Elsayed, M. Amin

#### Dr. Erfan & Bagedo General Hospital, Jeddah, Saudi Arabia

##### **Correspondence:** Abdelrahman Metwally Sr.

**Background:** Atrial fibrillation (AF) is the commonest sustained cardiac rhythm disorder and is responsible for substantial mortality and morbidity due to stroke, thrombo embolism, heart failure, reduced quality of life and impaired cognitive function. This arrhythmia is commonly seen in everyday practice. The most important threats patients with atrial fibrillation face are stroke and heart failure. Furthermore, quality of life is diminished due to AF symptoms as well as frequently associated cardiovascular diseases like hypertension, heart failure, coronary artery and valvular diseases. In the present study, the relationship between severity of mitral regurgitation (MR) and thrombo embolic risk in patient with AF was studied.

**Methods:** Forty five patients (21M/24F) 30patients with AF and MR referred to our cardio. dep., and 15 patients with atrial fibrillation without MR between Oct. 2012 and April 2013.Patients were subjected to full history taking, clinical evaluation, ECG., Transthoracic and transesophageal Echocardiographic (TEE) assessment was done. The severity of MR was assessed based on American Society of Echocardiography (ASE) guidelines. Multiple standard tomographic planes were imaged by (TEE) for assessment of left atrium, spontaneous echo contrast (SEC), left atrial appendage (LAA) empting velocities and LAA thrombi. Data were coded & entered using the statistical demo version of the graph Pad InStat, P value < 0.05 was considered statistically significant

**Results:** It was found that thrombo embolic risk has been shown to increase significantly in patients with AF without MR compared to patients with AF with MR. SEC is increased significantly in patients with mild MR than patients with moderate and severe MR and no significant changes between SEC grade in moderate and severe MR. The prevalence of LAA thrombus is increased in patients with mild MR than patients with severe MR. LAA empting velocity is deceased in patients without MR than patients with MR and decreased in patients with mild MR compared to patients with severe MR.

## A-6 The Role of Endothelial to Mesenchymal Transition (EndMT) in the Recovery from Heart Failure (HF)

### Hernan Gerardo Marcos-Abdala, Ana Sofia Cruz Solbes, Arvind Bhimaraj

#### Houston Methodist Research Institute, Houston, TX, USA

##### **Correspondence:** Hernan Gerardo Marcos-Abdala

**Background:** EndMT has been identified as a contributor to fibrosis in HF. We explore the role of this pathway in the reversal of HF in a murine model of recovery.

**Hypothesis:** EndMT is blocked, resulting in decreased fibrosis, increased contractility, and recovery from HF. Expression of EndMT signature genes is suppressed during recovery.

**Methods:** We have a unique mouse model of HF recovery with a non-ischemic heart failure phase induced by L-NAME and NaCL in drinking water and an Angiotensin II osmotic pump which creates HF in 5 weeks. We removed the injuring agents at 5 weeks and observed up to 19 weeks. We had 5 mice per group with 9 time points, including controls, HF weeks 3 and 5, and recovery weeks 7, 9, 11, 13, and 19. Echocardiography, heart weight, fibrosis content and RNA sequencing were performed at each time point.

**Results:** There is a complete reversal of fibrosis with a concomitant increase in ejection fraction in the mice after removal of injury by 19 weeks, compared to 5 weeks (peak HF) (p<.05). Of the 329 genes from the EMT database, 29 genes divided into 4 distinct patterns. For example, TGFB2 a known stimulant of EndMT, peaks expression by 5 weeks and gradually declines by week 9 while Lims1, a cell adhesion molecule on endothelial cells, is decreased during heart failure and reversed during recovery..

**Conclusion:** In our murine recovery model of heart failure we have found evidence suggesting an active role of EndMT in the pathology of HF which is then reversed during recovery, resulting in increased cardiac function. The modulation of this pathway could potentially promote recovery in HF.

## A-7 Predictors of response after CRT implantation in heart failure patients

### Bassam Hennawy, Haitham Badran, Sais Khaled, Hany Awadallah

#### Ain Shams University, Cairo, Egypt

##### **Correspondence:** Bassam Hennawy

**Background:** Current inclusion criteria may not be accurate enough to differentiate patients who will or will not respond to CRT. Other pathophysiologic factors such as HF etiology, LV dimensions and function, mitral regurgitation, LV dyssynchrony, position of LV pacing lead, and extent/location of myocardial scar have also shown to influence CRT response.

**Hypothesis:** This was a retrospective study that aimed at characterization of responders after CRT implantation, determining predictors of good or bad response to CRT.

**Methods:** This study included 50 patients with congestive heart failure refractory to optimum medical treatment who received CRT according to guidelines. All patients were subjected to thorough history taking, physical examination, ECG and echocardiographic study before and after the procedure and a fluoroscopic study to assess LV lead position.

**Results:** Responders to CRT were determined either clinically or according to echocardiographic study, clinical responders were 62% of the patients vs 60% echocardiographic responders. Predictors of good response to CRT were female gender (P=0.009), non-diabetics (P=0.006), non-dyslipidemics (P <0.001), higher NYHA class of the patient (P <0.001), non-ischemic cardiomyopathy (P=0.013), absence of paracardiac conditions (P=0.028), higher heart rate (P=0.017), wider QRS complex (P=0.007), lateral position of LV lead (P <0.001) and larger distance between RV and LV leads (P <0.001)

**Conclusion:** Ten predictors of response to CRT could be identified from this study apart from the current guidelines for CRT insertion.

## A-8 Lower Ventricular Myocardial Mitochondrial ROS Emission after Left Ventricular Unloading

### Elric Zweck, Daniel Scheiber, Julius B. Borger, Tomas Jelenik, Patrick Horn, Udo Boeken, Diyar Saeed, Malte Kelm, Michael Roden, Julia Szendroedi, Ralf Westenfeld

#### University Hospital Duesseldorf Department of Cardiology, Dusseldorf, Germany

##### **Correspondence:** Elric Zweck

**Background:** Impaired myocardial mitochondrial function and increased production of reactive oxygen species (ROS) have been suggested to contribute to ventricular dysfunction in heart failure. Therapeutic options are still rare. Recently, we showed more efficient mitochondria and lower ROS release in terminal heart failure patients with chronic left ventricular (LV) unloading compared to those without mechanical support. However, longitudinal data in individual patients of LV unloading effects on myocardial mitochondrial metabolism are lacking so far.

**Hypothesis:** We hypothesize that chronic LV unloading is associated with a reduction of myocardial mitochondrial ROS emissions.

**Methods:** In seven patients LV tissue specimens were acquired at time of implantation of an LV assist device and consecutively in the same patients at time of heart transplantation. We assessed LV myocardial mitochondrial hydrogen peroxide emission, oxidative capacity and coupling efficiency, as well as citrate synthase activity (CSA) as a marker of myocardial mitochondrial content.

**Results:** At time of heart transplantation, after an average LV unloading time of 174±70 days, myocardial ROS emission was 40% lower (0.92±0.11 vs. 0.55±0.11 pmol/(s*mg tissue), p<0.05). CSA did not differ between both time points (6.01±0.49 vs. 5.93±0.73 nmol/(s*mg protein), p=0.89), thus ROS emission normalized to CSA was 39% lower after LV unloading (0.14±0.01 vs. 0.09±0.02 (pmol H2O2*mg protein)/(nmol CSA*mg tissue), p<0.05). This indicates that ROS reduction is not a result of reduced mitochondrial content. Myocardial mitochondrial oxidative capacity and coupling efficiency did not change significantly before and after unloading, neither per milligram wet tissue (oxidative capacity: p=0.16; coupling efficiency: p=0.68), nor in proportion to mitochondrial content (oxidative capacity: p=0.57). However, the shorter the unloading duration, the more the mitochondrial coupling efficiency tended to increase (r=-0.71; p=0.09).

**Conclusion:** Our longitudinal data give first evidence for LV unloading-associated reduction of myocardial mitochondrial ROS emissions. This suggests protective effects of LV unloading on myocardial metabolic remodeling.

## A-9 Mortality in Infarct-Related Cardiogenic Shock Patients Treated with an Impella Microaxial Pump – Influence of Timing and Predicted Risk

### Andreas Schäfer^1^, Nikos Werner^2^, Jan-Thorben Sieweke^1^, Andreas Zietzer^2^, Maryna Masyuk^3^, Nanna Louise Junker Udesen^4^, Ralf Westenfeld^4^, Jacob Eifer Møller^4^

#### ^1^Hannover Medical School, Hannover, Germany; ^2^Universität Bonn, Bonn, Germany; ^3^University Hospital Düsseldorf, Dusseldorf, Germany; ^4^Odense University Hospital, Odense, Denmark

##### **Correspondence:** Andreas Schäfer

**Background:** Acute myocardial infarction (AMI)-related cardiogenic shock (CS) is associated with high mortality despite urgent revascularisation.

**Hypothesis:** Impella reduces mortality in patients with high predicted risk.

**Methods:** We analyzed data from 166 consecutive AMI-CS patients treated at four dedicated shock centers meeting the inclusion/exclusion criteria of the IABP-Shock II-trial (age 65±12 years), who received an Impella microaxial pump. Impella 2.5 was used in 39 patients (23%), Impella CP in 127 patients (77%). Mortality risk was calculated individually using IABP-Shock II-, CardShock- and SAVE-scores..

**Results:** Cardiac arrest had occurred in 68 patients (41%) prior to Impella implantation. Impella was implanted prior to PCI in 68 patients (41%).Observed vs. individually predicted mortality using CardShock-, Shock II-, and SAVE-scores was compared. Overall 30-day mortality was 43%. Mortality was higher in resuscitated patients (51% vs. 36%, p=0.0324) and when Impella was implanted post-PCI (Impella-pre-PCI: 29%, Impella-post-PCI: 51%, p=0.0130). The median CardShock-score was 5 predicting a 40% 12-days mortality (with only 28% resuscitated patients in CardShock), median Shock II-score was 3 predicting a 49% 30-days mortality, and median SAVE-score was -9 predicting 69% in-hospital mortality. In all used score systems observed mortality was lower than predicted mortality in those individuals with highest risk. Furthermore, mortality was significantly lower in patients who received Impella pre-PCI compared to after PCI.

**Conclusion:** In the absence of prospective trials, our retrospective analysis encourages the use of active mechanical circulatory support by Impella microaxial pumps in high-risk patients with AMI-CS undergoing PCI and supports the concept of early implantation prior to PCI. An adequate powered prospective trial, however, should assess the influence of active support devices on mortality in AMI-CS patients.

## A-10 Impella combined with veno-arterial extracorporeal membrane oxygenation effectively unloads the left ventricle with improving hemodynamics in a dog model of cardiogenic shock

### Genya Sunagawa, Keita Saku, Takuya Nishikawa, Takuya Akashi, Takuya Kishi, Hiroyuki Tsutsui, Kenji Sunagawa

#### Kyushu University, Fukuoka, Japan

##### **Correspondence:** Genya Sunagawa

**Background:** The combination of Impella and VA-ECMO, so called ECPELLA, is an essential strategy for patient with cardiogenic shock (CS). The pressure-volume area (PVA) of left ventricle (LV) per beat is proportional to LV oxygen consumption (MVO2). Impella decreases PVA depending on its support level. Contrary to this, VA-ECMO increases PVA, especially in LV dysfunction. In this study, we investigated how ECPELLA impacts on PVA and hemodynamics in CS.

**Methods:** In 5 mongrel dogs (15.1±0.3 kg), we created AMI by ligating coronary arteries. AMI lowered mean arterial pressure (MAP) below 70 mmHg in every dog. We placed Impella CP in LV and measured PV loops. We pumped blood from the central vein to the femoral artery and established ECMO. ECMO flow was fixed at 1.8 L/min. Impella flows were fixed at low (P1-2) and high (P4-6) levels. We compared hemodynamics and PVA under 6 conditions; Baseline, ECMO, Impella (low/high) and ECPELLA (ECMO with low/high Impella) in each dog. We also conducted simulation study (Matlab) and examined how changes in ECPELLA support level and LV function interacts in determining hemodynamics and PVA.

**Results:** Compared to Baseline, ECMO increased MAP (88±11 vs. 63±9 mmHg, p<0.01), and PVA (2088±357 vs. 1500±326 mmHg*ml, p<0.01). PVA in ECPELLA with low Impella was comparable to Baseline. In contrast, ECPELLA with high Impella markedly decreased PVA (258±182 vs. 1500±326 mmHg*ml, p<0.001) and LVEDP, and further increased MAP. Simulation study indicated that Impella support decreased the ECMO-increased PVA especially in depressed LV. Furthermore, ECPELLA made it possible to establish total LV unloading (zero PVA) with highest risk. Furthermore, mortality was significantly lower in patients who received Impella pre-PCI compared to after PCI.

**Conclusion:** The optimal use of ECPELLA unloads LV and improves hemodynamics in a dog with AMI-CS. The combination of ECMO and Impella, both of which are clinically available, may serve as a new strategy to achieve effective LV unloading with improving hemodynamics.

## A-11 Transcaval Access for the Emergency Delivery of Mechanical Circulatory Support in Cardiogenic Shock

### Majed Afana, Mahmoud Altawil, Mir Basir, Mohammad Alqarqaz, Khaldoon Alaswad, Marvin Eng, William W. O’Neill, Robert J. Lederman, Adam B. Greenbaum

#### ^1^Henry Ford Hospital. Detroit, MI, USA

##### **Correspondence:** Majed Afana

**Background:** Vascular access for the delivery of mechanical circulatory support (MCS) in patients with cardiogenic shock is often challenging due to peripheral arterial disease and vasoconstriction. Transcaval delivery of MCS may offer an alternative option. We describe the first series of patients in whom we implanted an Impella 5.0 device, without prior CT planning, through a percutaneous transcaval access route.

**Hypothesis:** We hypothesize that transcaval access for the delivery of higher-flow MCS in cardiogenic shock is a feasible alternative in patients with peripheral arterial disease or profound shock needing increased support.

**Methods:** Between December 2015 and June 2017, ten patients with progressive or refractory cardiogenic shock underwent transcaval Impella 5.0 implantation via a transcaval access. Demographic, clinical and procedural variables, along with in-hospital outcomes were collected.

**Results:** All ten patients underwent emergency implantation of the 7mm diameter Impella 5.0L device via transcaval access, without prior CT-based planning. Six were women, with median age of 55.5 years (range, 29 – 69). Cardiogenic shock was attributed to idiopathic non-ischemic cardiomyopathy (n=4), myocarditis (n=2), ischemic cardiomyopathy (n=2), post-heart transplant rejection (n=1), and unknown etiology (n=1). Median duration of support was 92.1 hours (range, 21.2 – 165.4). Seven (70%) survived to device explant, with six (60%) surviving to transcaval access port closure and discharge. One transcaval sheath left in place for hemodynamic monitoring during a planned terminal wean in the setting of progressive clinical decline. Among survivors to discharge, five recovered heart function and one underwent left ventricular assist device as destination therapy.

**Conclusion:** Transcaval access for the delivery of MCS is a feasible alternative for emergency non-surgical implantation of the Impella 5.0 device in patients with peripheral arterial disease or those with profound cardiogenic shock. This approach allows earlier institution and longer duration of higher-flow MCS, and may enable a bridge-to-recovery or bridge-to-destination strategy.

## A-12 Reversal of genetic determinants of heart failure during cardiac recovery in a unique non-ischemic mouse model

### Arvind Bhimaraj, Guangu Wang, Hernan Marcos Abdala, Keith Youker, Guillermo Torre, Kaifu Chen, John Cooke

#### Houston Methodist Hospital, Houston, TX, USA

##### **Correspondence:** Arvind Bhimaraj

**Background:** Mechanisms of cardiac recovery are not well elucidated. Using a non-ischemic mouse model of HF that has NaCl and L-NAME to cause injury we have established a recovery model to study the same in a controlled setting.

**Hypothesis:** Genetic recovery parallels morphological recovery in a non-ischemic mouse model of heart failure recovery.

**Methods:** We established a complete phenotypic recovery of heart failure when 3-month old C56 male mice are induced with heart failure (using an angiotensin II pump and L-NAME) followed by removal of the inducing agents at 5 weeks followed by observation till Week 19.

**Results:** Morphological (Ejection fraction, heart weight) and histological (fibrosis content and myocyte size) criteria reflective of heart failure peaked at Week 5 and normalized to baseline after removal of injury. Heat maps of global gene expression were distinctly different at Week 5 HF arm compared to Week 5 controls and revert back to a pattern similar to Week 5 controls at 19 weeks. Comparison between 5 week HF and control revealed 1376 up regulated and 3188 down regulated genes while comparison of Week 19 to control revealed only 259 up regulated and 31 down regulated genes. Of the 254 genes in hypertrophy pathway 58 clustered into 4 specific patterns of significant change over the period of heart failure induction and recovery. Similarly of the 86 genes in the fibrosis pathway, 22 cohorted into 3 patterns.

**Conclusion:** We have established a mouse model of heart failure recovery confirmed not just by phenotypic markers but also by genetic expression. There are specific patterns of change of certain genes which could reveal relevant pathways for future therapeutic modulation.

## A-13 Smart Impella unloading in the acute phase of MI markedly reduces infarct size and prevents LV dysfunction in the long term

### Kazuhiro Kamada, Keita Saku, Genya Sunagawa, Takuya Nishikawa, Kenji Sunagawa

#### Kyushu University, Fukuoka, Japan

##### **Correspondence:** Kazuhiro Kamada

**Background:** Recent studies have shown that maximum LV unloading by Impella in AMI markedly reduces MI size and prevents subsequent LV dysfunction via the maximum reduction of myocardial oxygen consumption (MVO2). However, difficulties associated with manual control of Impella make its clinical applications impractical, in particular, under compromised AMI hemodynamics. To explore its clinical applicability, we developed Smart-Impella (S-Impella) algorithm to materialize automated control of Impella to maximally unload LV and evaluated its performance in AMI model dogs.

**Methods:** S-Impella algorithm controls the instantaneous rotational speed of Impella to decouple LVP from arterial hemodynamics and to maximally reduce MVO2 by incorporating both LVP and AP in feedback loops. In 8 dogs, we created MI for 180 min and then reperfused. We introduced S-Impella from 60 minutes after the creation of ischemia to 60 min after reperfusion (I/R). Four weeks after I/R, we compared LV function and infarct size between S-Impella (n=4) and no-treated (Control, n=4) groups. Patient hemodynamic measures were evaluated as secondary outcomes.

**Results:** During the acute phase of MI, S-Impella adjusted LVSP at 44.1±9.7 mmHg while keeping LVEDP (4.6±1.2 mmHg) and mean AP (103±13 mmHg) at normal levels. Four weeks after I/R, S-Impella significantly lowered LV end-diastolic pressure (6.2±9.7 vs. 11.5±2.0 mmHg, p<0.05), increased LV end-systolic elastance (20.5±5.2 vs. 6.3±2.5 mmHg/ml, p<0.001), and decreased serum NT pro-BNP level (2198±1059 vs. 3558±778 pg/ml, p<0.05) compared to Control. Histological study indicated that S-Impella markedly reduced MI size in the chronic phase (4.9±1.5 vs. 9.6±2.6%, normalized by LV area; p<0.05).

**Conclusion:** In the acute phase, S-Impella stably unloaded LV and stabilized compromised hemodynamics. In the chronic phase, S-Impella prevented the worsening of LV dysfunction via the reduction of MI size. S-Impella, which helps eliminate the impediment of acute unloading under compromised hemodynamics, may serve as a novel and powerful therapeutic strategy for AMI.

## A-14 Effects of Cumulative Volume Status On Outcomes of Cardiogenic Shock

### Padmaraj Duvvuri, David Wolfe, Muhammad Said, Elizabeth Rowland, Sandeep Banga, Ekanka Mukhopadhyay, Sudhir Mungee, Chetan Bhardwaj

#### OSF St. Francis Medical Center. Peoria, IL, USA

##### **Correspondence:** Padmaraj Duvvuri

**Background:** Strict Input and output are uniformly recorded in the cardiovascular intensive care unit. The effects of cumulative volume as load on the heart is not completely understood

**Hypothesis:** Increased cumulative volume status is associated with poor outcomes, namely higher adverse safety outcomes (ASO) (including: re-infarction, VT/VF, need for new dialysis, stroke, and blood transfusion), length of stay (LOS), and mortality.

**Methods:** Retrospective chart study of patients presenting with cardiogenic shock due to acute coronary syndrome during calendar years 2015 through 2017 was done. Demographic, clinical presentation, laboratory and cardiac catheterization variables were collected. Outcomes such as LOS, ASO and mortality were collected.

**Results:** 173 patients were included in the study. 94 (54%) patients survived the hospital course. Mean age of the patient presenting with cardiogenic shock was 66.11 years. Mechanical circulatory support was utilized in 67 patients (54 IABP and 13 Impella®). Invasive monitoring with pulmonary artery catheter and arterial line was used in 39 and 112 patients respectively. Strict Ins and Outs were measured in all patients. Difference in cumulative volume was statistically significant in patients who survived (-293.78 mL) vs who died (4,292.04 mL), p < .001. Increasing cumulative volume was also associated with increasing mortality (1.03 times higher, per mL increase in cumulative volume). Similarly, patients with ASO events (2,414.00 mL) had higher cumulative volumes compared with those who did not (-240.15 mL), p < .001. No significant relationship between cumulative fluid balance and length of stay was found.

**Conclusion:** Higher cumulative volume is associated with higher mortality and adverse outcomes in hospital events. There no significant effect on hospital length of stay was found. Cumulative fluid balance is potentially a surrogate of cardiac load. After acute unloading in cardiac catheterization laboratory, continued unloading of volume through the hospital course may be necessary to decrease in-hospital mortality and adverse outcomes.

## A-15 Mechanical unloading exacerbates fibrosis in control rat hearts and after pressure overload

### Andreas Schaefer, Yvonne Schneeberger, Steven Schulz, Susanne Krasemann, Tessa Werner, Angelika Piasecki, Grit Höppner, Kristina Lorenz, David Wieczorek, Alexander P Schwoerer, Thomas Eschenhagen, Heimo Ehmke, Hermann Reichenspurner, Justus Stenzig, Friederike Cuello

#### University Heart Center, Hamburg, Germany

##### **Correspondence:** Andreas Schaefer

**Background:** Mechanical unloading (MU) by implantation of left ventricular assist devices (LVAD) has become clinical routine. This procedure has been shown to reverse cardiac pathological remodeling, with the underlying molecular mechanisms incompletely understood. Most studies thus far were performed in non-standardized human specimens or MU of healthy animal hearts. Our study investigates whether fibrotic remodeling differs between healthy hearts or hearts subjected to standardized pathological pressure overload by transverse aortic constriction (TAC) prior to MU.

**Methods:** Rats underwent sham or TAC surgery. Disease progression was monitored by echocardiography prior to MU by heterotopic heart transplantation (hHTx/MU). Hearts after TAC or TAC combined with hHTx/MU were removed and analyzed by histology, western immunoblot and gene expression analysis.

**Results:** TAC surgery resulted in cardiac hypertrophy and impaired cardiac function. TAC hearts revealed significantly enhanced cardiac myocyte diameter and mild fibrosis. Expression of pro-hypertrophic genes after TAC was higher compared to hearts after hHTx/MU. Whilst cardiac myocyte cell diameter regressed to the level of sham-operated controls in all hearts subjected to hHTx/MU, fibrotic remodeling was significantly exacerbated. Transcription of pro-fibrotic and apoptosis-related genes was markedly augmented in all hearts after hHTx/MU. Sarcomeric proteins involved in excitation-contraction coupling displayed significantly lower phosphorylation levels after TAC and significantly reduced total protein levels after hHTx/MU.

**Conclusion:** Exacerbation of myocardial fibrosis, cardiac myocyte atrophy and loss of sarcomeric proteins was observed independently of the disease state of the unloaded hearts. These results may help to explain the clinical experience with low rates of LVAD removal due to lack of myocardial recovery.

## A-16 The LV global longitudinal strain pattern is superior indicator to predict beneficial effect of IABP in patients with STEMI

### Surenjav Chimed, Dashdemberel Khatanbaatar, Batbold Bayaraa, Uyanga Ganbat

#### Second General Hospital, Ulaanbaatar, Mongolia

##### **Correspondence:** Surenjav Chimed

**Background:** The LV mechanical support is crucial in management of patient with STEMI who had poor cardiac contractility. The IABP is most frequently using method for acute cardiac unloading in STEMI. However, indicators which can predict beneficial effect of IABP in patients with STEMI are still unclear.

**Hypothesis:** We hypothesized that LV GLS is superior indicator to predict beneficial effect of IABP in patients with STEMI.

**Methods:** In this study, we selected patients with STEMI who treated by primary PCI. The IABP was choice of mechanical circulatory support in those patients. The LV global longitudinal strain (GLS) used to assess LV recovery during hospitalization. The 30-day mortality was chosen for primary endpoint.

**Results:** A total of 65 patients with STEMI who treated by primary PCI and implanted IABP were included (mean age 63±14, 78% male). The 30-day mortality was occurred in 24.6% of patients (n=16). LV GLS was significantly different between patients who survived and died within 30-days (-12.9±4.3% vs. -7.9±3.8%, p<0.001). In Cox regression, peak TnI level (HR=1.003. 95% CI 1.001-1.005, p<0.01), GFR (HR=0.96, 95% CI 0.94-0.98, p<0.001), Ee’ ratio (HR=1.05, 95% CI 1.02-1.08, p<0.01) and LV GLS (HR=1.23, 95% CI 1.10-1.37, p<0.001) were significantly associated with 30-day mortality. After adjustment of above mentioned variables, LV GLS (HR=1.27, 95% CI 1.09-1.48, p<0.004) and GFR (HR=0.97, 95% CI 0.94-0.99, p<0.05) were independently associated with 30-day mortality. In ROC curve estimation, LV GLS showed good predictive capacity (AUC=0.80, 95% CI 0.69-0.92, p<0.001) and cut-off value of LV GLS which best differentiate patients who survived was -11.5% (sensitivity 87.5% and specificity 65.3%).

**Conclusion:** The LV GLS is independently associated with 30-day mortality in patients with STEMI who implanted IABP. It was best indicator to determine beneficial effect of IABP for 30-day mortality.

## A-17 Use of Percutaneous Right Ventricular Assist Device in Patients with Acute Right Ventricular Failure following the Left Ventricular Assist Device Implantation

### Asim Mohammed, Sakthi Sundararajan, Michael Cain, Bernice Badu, Lucian Durham, David Joyce

#### Medical College of Wisconsin, Milwaukee, WI, USA

##### **Correspondence:** Asim Mohammed

**Background:** Right ventricular (RV) failure is a frequent complication of left ventricular assist device (LVAD) implantation in patients with end-stage heart failure, impacting 20% of patients. RV failure post LVAD is associated with 40% 1-year mortality. Percutaneous support can be used in this scenario, however data supporting this approach is lacking. .

**Methods:** A retrospective review was conducted of all patients who developed severe RV failure requiring percutaneous right ventricular assist device (RVAD) following durable LVAD implant at our academic institution between 01/01/2017 and 07/31/2018. Patient demographics were reviewed in addition to key hemodynamic and laboratory values were collected pre-RVAD implantation (immediately prior to cannulation) and at the time of explant (post-RVAD). Pre-RVAD and post-RVAD data was compared using paired T tests. Survival at 30 days or at date of hospital discharge (whichever was longer) was considered as the primary outcome. Patient hemodynamic measures were evaluated as secondary outcomes.

**Results:** Of 41 patients who received LVAD, 10 (24%) developed severe RV failure postoperatively. Baseline characteristics include age 51±14.5 years, with 30% females. Incidence of ischemic cardiomyopathy was 20%. Most patients received LVAD as destination therapy (80%). RVAD support via percutaneous cannulation was maintained for a median duration of 15.5 days (Range – 7 to 23 days). Survival at 30 days or to hospital discharge was 100%. RVAD support was successfully weaned in 9 patients (90%), while the remaining 1 patient underwent cardiac transplantation. We observed a reduction in central venous pressure from 17.2±4.5 mm (Hg) to 11±3.1 mm (Hg), p=0.004 and an increase in LVAD flow from 4.5±0.8 l/min to 5.1±0.4 l/min, p=0.018. There was a trend towards increase in mixed venous oxygen saturation from 61.5±11.1% to 67.0±7.0%, p=0.224.

**Conclusion:** Percutaneous RVAD cannulation for treatment of RV failure after LVAD implantation provides superior early postoperative survival than traditional management techniques. These findings support broader implementation of this technique to further characterize its benefit.

## A-18 Hemolysis in Impella®-support during cardiogenic shock relates to the device-suction events

### Sandra Bueter, Jean-Marc Haurand, Malte Kelm, Ralf Westenfeld, Patrick Horn

#### University Hospital Duesseldorf, Department of Cardiology, Dusseldorf, Germany

##### **Correspondence:** Sandra Bueter

**Background:** Hemolysis is a common complication in patients with cardiogenic shock (CS) treated with Impella® support which might lead to acute renal injury, hyperkalemia and the need of blood transfusion. Up to now it is not known which factor triggers hemolysis in vivo and how the occurrence may be prevented.

**Hypothesis:** The aim of this study was to investigate whether suction of the device pump triggers hemolysis in patients with CS and Impella® support.

**Methods:** In this retrospective study we analyzed 34 patients with CS treated by Impella® device for at least 24h. Hemolysis was evaluated by decrease in hemoglobin and haptoglobin plus increase in lactate dehydrogenase and bilirubin. Suction of the pump device was counted as the number and the duration of suction alarm events during Impella® support by analyzing the complete performance data of the device console.

**Results:** 22 out of 34 patients suffered hemolysis during Impella® support. The groups (hemolysis vs. non-hemolysis) did not differ between age, SOFA Score, APACHE II Score, basal lactate value, comorbidities or prior resuscitation. There was no difference in rate of hemolysis between Impella 2.5l® and Impella CP®

**Conclusion:** Hemolysis in patients with Impella® support is associated with device suction. Future trials should prospectively evaluate whether different strategies for Impella® positioning at various anatomies or adaptation of pump-speed and volume status may decrease hemolysis.

## A-19 Putting LVOT VTI Impell-spective II: A Retrospective Study of LVOT VTI as a Weaning Parameter for Shock in Intervened-on AMI Patients

### Ahmad Z. Turk, Kyle Gobeil, Anis John Kadado, Fotis Katsikeris, Daniel T. Engelman, Evan Y. Lau, Jaime Hernandez Montfort

#### Baystate Medical Center, Springfield, MA, USA

##### **Correspondence:** Ahmad Z. Turk

**Background:** There is absence of robust evidence-based recommendations describing use and clinical impact of daily echocardiographic evaluation for mechanically unloaded patients supported with temporary micro-axial flow pumps (Impella).

**Hypothesis:** In patients presenting with cardiogenic shock secondary to acute myocardial infarction (LH AMI-CS) requiring prolonged Impella support after revascularization, adjunct protocolized bedside echocardiographic weaning evaluation offers a reliable surrogate of myocardial performance prior to explant.

**Methods:** Our description included retrospective evaluation of a database of patients supported with Impella device (CP/5.0) at Baystate Medical Center Cardiac Intensive Care Unit from 2014 to 2017. Inclusion criteria consisted of having 1) AMI with percutaneous or surgical revascularization, 2) clinical and hemodynamic cardiogenic shock and 3) prolonged Impella support requiring ICU level of care. Exclusion criteria included 1) Non-obstructive coronary anatomy, 2) Intraprocedural Impella removal after intervention, 3) no revascularization, 4) no evaluation of LVOT VTI on serial echocardiograms while on support. Serial LVOT VTI and corresponding CI were recorded spanning the implant-explant time. We plotted on 2 correlation graphs the results as well as the change in LVOT VTI/Cardiac Index relative to their initial values after implant.

**Results:** A total of 38 VTI/CI results showed a positive regression of 0.67. Relative to the initial values, 26 relative VTI/CI results showed a positive regression of 0.58 (p-value=0.0019), suggestive of a strong correlation between the assessed LVOT VTI, and the calculated CI.

**Conclusion:** We provide a real-world standardized method that objectively focuses on routine evaluation of LVOT VTI as a surrogate of myocardial performance after AMI-CS s/p revascularization requiring prolonged mechanical unloading. Further research is required to further demonstrate the safety and feasibility of this method of surveillance which might impact daily clinical decision making of patients supported with percutaneous micro-axial flow pumps in distinct clinical scenarios.

## A-20 Mechanical circulatory support with Impella® in patients with non-acute myocardial infarction related cardiogenic shock

### Jean-Marc Haurand, Sandra Büter, Christian Jung, Malte Kelm, Ralf Westenfeld, Patrick Horn

#### University Hospital Dusseldorf, Department of Cardiology, Dusseldorf, Germany

##### **Correspondence:** Jean-Marc Haurand

**Background:** Mechanical circulatory support with the Impella® pump is used to hemodynamically stabilize patients with cardiogenic shock (CS) caused by acute myocardial infarction (AMI) until cardiac function has recovered after coronary revascularization. Whether Impella® support is effective to stabilize patients with non-AMI-related CS is not well investigated..

**Hypothesis:** MCS with Impella® is likely effective to stabilize patients with non-AMI-related CS compared to patients with AMI-related CS.

**Methods:** We retrospectively analyzed 106 patients with CS and Impella® support in the years from 2011 to 2018. 36 patients suffered from non-AMI CS and 70 patients from AMI-related CS. The efficacy to stabilize the patient was assessed by laboratory values such as lactate, hemodynamic parameters and clinical scores. The difference in mortality was calculated with the Log-Rank-Test, comparing Kaplan-Meier curves.

**Results:** Patients with non-AMI-related CS and patients with AMI-related CS were severely ill, and did not differ in APACHE II, SOFA score and cardiac index. In both groups, lactate levels decreased equally in the first 48h during Impella® support, from 4.1 ± 0.52 mmol/l to 1.7 ± 0.2 mmol/l (p =< 0.001) in the non-AMI group and from 3.6 ± 0.48 mmol/l to 2.2 ± 0.72 mmol/l (p = 0.025) in the AMI group. There was a trend to a lower thirty-day mortality in the non-AMI group compared to the AMI group (47 % vs. 57 %, p = 0.067). An upgrade to long-term ventricular assist devices was performed in 22 % of the non-AMI patients and in 6 % of the AMI patients (p = 0.011).

**Conclusion:** Impella® support was effective to hemodynamically stabilize patients with non-AMI-related CS. Therefore, MCS with Impella® is useful in these patients as a bridge to recovery or a bridge to upgrade for long-term left ventricular support.

## A-21 Left Ventricular Unloading during Peripheral Extracorporeal Membrane Oxygenator Support: A Bridge to Life in Profound Cardiogenic Shock

### Matteo Attisani, Lodo V, Baronetto A, Simonato E, Boffini M, Ricci D, Centofanti P, Rinaldi M

#### University of Turin, Turin, Italy

##### **Correspondence:** Matteo Attisani

**Background:** A limit of peripheral veno-arterial Extracorporeal Membrane Oxigenator (VA-ECMO) is the inadequate unloading of the left ventricle. The increase of end-diastolic pressure reduces the possibility of a recovery and may cause severe pulmonary edema.

**Hypothesis:** In this study, we evaluate our results after implantation of VA-ECMO and Transapical Left Ventricular Vent (TLVV) as a bridge to recovery, heart transplantation or long-term left ventricular assit devices (LVAD).

**Methods:** From 2011 to 2018, 50 consecutive patients with pre-cardiotomic profound cardiogenic shock were supported by peripheral VA-ECMO as bridge to decision. In all cases, TLVV was implanted after a mean period of 12.2 ± 3.4 hours through a left mini-thoracotomy and connected to the venous inflow line of the VA-ECMO.

**Results:** In-hospital mortality was 50.0% (25/50). In all patients, hemodynamics improved after TLVV implantation with an increased cardiac output, mixed venous saturation and a significant reduced heart filling pressures (p < .05). Recovery of the cardiac function was observed in 18 patients (18/50; 36%). Five patients were transplanted (5/50; 10%) and six patients (6/50; 12%) underwent LVAD implantation as destination therapy or bridge to candidacy, 10 of these patients were discharged from the hospital in good clinical conditions.

**Conclusion:** In these critical patients, systematic TLVV improved hemodynamic seemed to provide better in hospital survival and chance of recovery, compared to VA-ECMO results in the treatment of cardiogenic shock reported in the literature. TLVV is a viable alternative to standard VA-ECMO to identify the appropriate long-term strategy (heart transplantation or long-term VAD) reducing the risk of treatment failure. A larger and multicenter experience is mandatory to validate these hypothesis.

## A-22 Electrophysiological remodeling induced by mechanical unloading is rescued by phospholamban deletion

### Heimo Ehmke, Sumi Westhofen, Leonie Dreher, Helga Vitzhum, Hermann Reichenspurner, Alexander P. Schwoerer

#### University Medical Center Hamburg-Eppendorf. Hamburg, Germany

##### **Correspondence:** Heimo Ehmke

**Background:** Mechanical unloading of the left ventricle is associated with an increased likelihood of potentially life-threatening ventricular arrhythmias. Alterations in myocardial Ca2+ cycling probably contribute to the underlying electrophysiological remodeling, but the precise mechanisms are unknown. A major consequence of mechanical unloading is a marked upregulation of phospholamban (PLN) expression and activation by dephosphorylation, leading to a shift of Ca2+ cycling from the sarcoplasmic reticulum to the plasma membrane.

**Hypothesis:** We investigated the hypothesis that a prevention of PLN activation attenuates the electrophysiological remodeling caused by cardiac mechanical unloading.

**Methods:** Unloading was induced by heterotopic heart transplantation in littermate wildtype (WT) and PLN deficient (PLN-/-) mice, with orthotopic hearts serving as controls. Cardiac electrophysiological and molecular remodeling were analyzed by measuring field potentials in cardiac slices with microelectrode array, and by quantitative real-time PCR and Western blotting.

**Results:** Unloading of the left ventricle for 2 weeks increased field potential (FP) duration by 28% (from 96+/-3 ms, n=30 to 123+/-3 ms, n=18; P<0.001) in WT mice. This prolongation was completely absent in cardiac slices from PLN-/- mice (83+/-1 ms, n=49 vs. 81+/-3 ms, n=18). In contrast, changes in gene expression were much more pronounced in PLN-/- mice which exhibited markedly reduced expression levels of the voltage-gated L-type calcium channel (Cav1.2: -55%) and of the molecular components underlying the transient outward K+ current Ito (Kv4.2: -75%; Kv4.3: -46%; KChIP2: -80%).

**Conclusion:** Genetic deletion of PLN rescues the electrophysiological remodeling induced by mechanical unloading. Our findings suggest PLN inhibition as a new therapeutic option to prevent cardiac arrhythmias associated with mechanical unloading.

## A-23 Mechanical unloading as a novel treatment option for chronic inflammatory cardiomyopathy – a mode of action study

### Frank Spillmann^1^, Sophie Van Linthout^1^, Oliver Klein^1^, Thomas Mairinger^1^, Evgenij V. Potapov^1^, Daniel Burkhoff^2^, Burkert Pieske^1^, Carsten Tschöpe^1^

#### ^1^Charité - Universitaetsmedizin Berlin, Berlin, Germany; ^2^Columbia University Medical Center, New York, NY, USA

##### **Correspondence:** Frank Spillmann

**Background:** Hemodynamic load induces cardiac remodeling via mechanotransduction pathways, which can further trigger inflammatory responses. Evidence form left ventricle (LV) assistant devices in chronic heart failure (HF) patients indicate that mechanical unloading can lead to reverse remodeling based on anti-fibrotic and anti-inflammatory mechanisms. Particularly in a cardiac inflammatory disorder as inflammatory cardiomyopathy, a therapeutic strategy is required, which in addition to providing adequate circulatory support, unloads the LV, decreases cardiac wall stress, and mitigates inflammatory responses. Axial flow pumps like the Impella systems directly unload the LV throughout the cardiac cycle, decreasing total mechanical work, while lowering wall stress an improving subendocardial coronary blood flow.

**Hypothesis:** We hypothesized that prolonged unloading via a percutaneously implanted Impella-pump system over 4 weeks is a strategy to improve HF due to chronic inflammatory cardiomyopathy and investigated the mode-of-action.

**Methods:** An Impella was implanted in a patient with inflammatory cardiomyopathy and pre-cardiogenic shock despite immunosuppression. Endomyocardial biopsies (EMB) were taken over time for molecular biology and immunohistochemistry purposes.

**Results:** Impella implantation led to an improvement in LV-EF (from 15 to 53%) and a reduction in NT-pro-BNP levels (from 8700 to 690 pg/ml). During unloading, cardiac immune cell presence (CD45RO, Mac, LFA-1 and CD3) and LV expression of the adhesion molecules ICAM-1 and VCAM-1 decreased. Concomitantly, the expression of the integrins α1, 5, 6, 10 and ß6 was 2.0-, 2.0, 3.9-, 6.6- and 1.5-fold downregulated during unloading, respectively. However, those effects were abrogated after removal of the LV-Impella 5.0 support despite continuation of immunotherapy, suggesting a primary “unloading”-dependent mechanism. Imaging mass spectrometry further revealed a change in total protein muster including the modulation in extracellular matrix production as shown by a decrease in collagen α-2VI and vimentin expression during unloading.

**Conclusion:** Prolonged unloading with an Impella device offers a circulatory support with additional disease modifying effects important for bridge-to-recovery in patients with inflammatory cardiomyopathy.

## A-24 The interest of tissue doppler imaging in the weaning of veno-arterial extracorporeal membrane oxygenation

### Nadia Ouazani, Yasuhiro Shudo, Charles Hill, Joe Hsu, JefferyTeuteberg

#### Stanford University, Palo Alto, CA, USA

##### **Correspondence:** Nadia Ouazani

**Background:** VA ECMO use has exploded worldwide these last 2 decades. However, many shadowed areas remain in the setting of ECMO. Notably, there is no clearly guideline in the process of ECMO weaning. One retrospective study found out that LVEF>20-25%, VTI>10cm/sec and TDSa>6 cm/sec were predictive of a successful ECMO withdrawal. Our aim is to confirm the previous findings and to identify other predictor echocardiographic parameters of a successful ECMO removal without requiring any further MCS.

**Hypothesis:**

**Methods:** This prospective study is performed in our ICU, with the approval of our IRB. Each patient hospitalized in ICU under VA ECMO is eligible except if he has a severe mitral disease or mitral repair. A TTE is performed daily. A weaning trial is realized in patient with stable hemodynamic conditions: MAP above 60mmhg, presence of an arterial pulsatility and no issue with pulmonary blood oxygenation. The weaning is stopped if MAP decreases below 60mmHg. The conventional clinical parameters are recorded. The echocardiographic indices include: LVEF, aortic time-velocity integral (VTI), systolic tissue doppler imaging (TDI) on lateral (SaL) and septal (SaS) mitral annulus, early diastolic TDI on lateral (EaL) and septal (EaS) mitral annulus and global longitudinal strain on LV (GLS LV) in 4 cavities. The ECMO removal is considered if LVEF>25% and VTI>10 cm/sec.

**Results:** We included 17 patients in the study, of whom 4 myocardial infarction. The median age was 48y, the SAPScore II at 38. Among them, 4 died from MOF, 1 was unable to support the weaning trial and benefited a VAD. So 12 underwent a complete weaning trial of whom 9 were finally weaned (Weaned group), 1 decreased his MAP below 60 mmHg after the trial at half ECMO flow requiring a VAD immediately after decanulation and 2 with a delay of few days after ECMO removal (VAD group, n=3). During the ramp, the LVEF, VTI, GLS LV, and TDI SaS increased significantly while decreasing the ECMO flow. Only the TDI SaL and the early diastolic TDI remained insensitive to flow variations (p>0,05). At the lowest ECMO flow, there was no significant difference between the 2 groups in VTI, LVEF, EaL, EaS, GLS LV indices, whereas TDI SaL and SaS were higher in Weaned group than VAD group, respectively 8.75 against 5.43cm/sec and 8.1 against 4.9cm/sec, (p<0,05).

**Conclusion:** Systolic TDI may be a reliable predictor of successful ECMO weaning, in particular TDI SaL, which appears independent of loading variations.

## A-25 Weaning of temporary percutaneous LV mechanical circulatory support in cardiogenic shock

### Theodore Schreiber, Nimrod Blank, Amir Kaki

#### Detroit Medical Center Heart Hospital. Detroit, MI, USA

##### **Correspondence:** Theodore Schreiber

**Background:** Acute cardiogenic shock, CS, is associated with high mortality rates. While CS diagnosis criteria are well established and are fundamental in guiding pVAD use, the decision of the pVAD weaning is of extreme challenge and much less supported with established data. Cardiac power index, CPO, introduced at the SHOCK trial, was shown to be the single best indicator of cardiovascular performance. The National CS Initiative, CSI, suggests parameter for pVAD weaning decision.

**Hypothesis:** To present our experience and strategy toward successful pVAD weaning for patients recovering from CS.

**Methods:** The Detroit Medical Center, DMC, a tertiary center and part of the Natonal CSI is highly experienced in treating CS. We retrospectively analyzed the data of 89 consecutive patients who were treated with pVAD (Impella CP) for CS / severe decompensating HF and were assessed with cardiovascular recovery into pVAD extraction, monitoring PA/systemic pressures, cardiac output, cardiac power output, echocardiography, together with systematic vital organ assessment.

**Results:** 89 patients (58% CS, 33% LV unloading, 5% post cardiac arrest, 3% others) were treated and weaned of pVAD support. Overall, 74 (83%) patients survived to CCU discharge. Of the 15 (17%) patients who did not survived, in 10 patients pVAD extraction was done despite non optimal hemodynamic recovery which did not meet our criteria for successful pVAD weaning. In all of this patients pVAD extraction was done for irretraceable sepsis or bleeding possibly caused or aggravated by the artificial pVAD hardware and the requirement for aggressive anti-coagulation. In 5 patients; despite favorable hemodynamic and cardiovascular performance, hemodynamic collapse followed pVAD extraction with catastrophic results. .

**Conclusion:** Multimodality assessment with focus over cardiovascular hemodynamic performance is necessary to assure high success rate in pVAD weaning for patient recovering CS.

## A-26 Left ventricular unloading with Impella causes cardiac metabolic remodeling with a significant reduction in myocardial glucose consumption and lactate production

### Carlos G. Santos-Gallego, Olympia Bikou, Kelly Yamada, Shin Watanabe, Belen Picatoste, Alvaro Garcia-Ropero, Juan Badimon, Roger Hajjar, Kiyotake Ishikawa

#### Mount Sinai Hospital, New York, NY, USA

##### **Correspondence:** Carlos G. Santos-Gallego

**Background:** Normal myocardium produces ATP mainly through free fatty acids (FFA) oxidation. However, during HF, there is a metabolic switch with the heart predominantly consuming glucose (which produces less ATP but also requires less oxygen) and even producing lactate (as a result of the anaerobic metabolism of glucose). The effect of acute LV unloading on myocardial metabolic remodeling has not yet been studied.

**Hypothesis:** We hypothesized that acute LV unloading using Impella would reduce myocardial glucose consumption and lactate production (thus improving adverse metabolic remodeling), as the ATP requirements of the unloaded LV are diminished.

**Methods:** Simultaneous catheterization of LAD and coronary sinus was performed to measure transmyocardial gradient (TG) of the different metabolites. We studied three groups: 1) sham animals, 2) 1 week post-MI before and after 2 hours of LV unloading (Impella, IMP), 3) 1 week post-MI before and after 2 hours of LV overloading (severe aortic regurgitation, AR)..

**Results:** Sham animals showed myocardial FFA consumption (TG 94±27μmol/L) with low utilization of both glucose (TG 112±45μmol/L) and only mild lactate consumption (TG 13±18μmol/L). HF animals exhibited reduced FFA consumption, increased glucose utilization and raised lactate production. LV overloading exacerbated myocardial glucose consumption (TG 546±229 preAR vs 1081±544μmol/L postAR, p<0.05) and lactate production (TG -89±24 preAR vs -177±47μmol/L postAR, p<0.05). LV unloading reduced both myocardial glucose consumption (TG 640±127 preIMP vs 180±93μmol/L postIMP, p<0.05) and lactate production (TG -83±59 preIMP vs 6±11μmol/L postIMP, p<0.05). No difference was found in myocardial uptake of FFA or ketones.

**Conclusion:** Failing hearts show a metabolic remodeling with reduction of FFA consumption and increase both in glucose utilization and lactate production. LV overloading aggravates this metabolic shift. LV unloading ameliorates this metabolic switch as both myocardial glucose consumption and myocardial lactate production are reduced. These data indicate that ventricular unloading in HF is able to acutely and rapidly shift metabolic substrate utilization.

## A-27 The right ventricular function is independent predictor of 30-day mortality in patients with STEMI complicated by cardiogenic shock: Is that RV is target of acute unloading?

### Punsaldulam Boldbayar, Dashdemberel Khatanbaatar, Batbold Bayaraa, Surenjav Chimed

#### Institute of Medical Sciences, Ulaanbaatar, Mongolia

##### **Correspondence:** Punsaldulam Boldbayar

**Background:** Cardiogenic shock (CS) is challenging condition in patients with STEMI who treated by primary PCI. The role of LV function abnormality is well studied in cardiogenic shock patients and conventional mechanical circulatory support (MCS) devices are mainly designated to unload LV pre and after load. However, role of RV in patients with CS complicating STEMI is not clear.

**Hypothesis:**. In this study, we aimed to reveal the role of RV function in patients with STEMI complicated by CS.

**Methods:** We selected cardiogenic shock patients with STEMI who treated by primary PCI. The RV function was evaluated by speckle-tracking derived strain parameter. The primary endpoint was 30-day mortality after index STEMI.

**Results:** A total of 51 patients with CS were included in this study (mean age 64±14, 76% male). During follow-up, occurrence of 30-day mortality was 27.5% (n=14). RV global strain (-15.7±4.6 vs. -11.5±6.1, p<0.05) significantly different between patients who survived and died. Univariable Cox regression analysis was revealed that previous CHF (HR=18.6, 95% CI 3.34-103.3, p<0.001), peak TnI level (HR=1.003, 95% CI 1.001-1.005, p<0.01), glomerular filtration rate (HR=0.97, 95% CI 0.95-0.99, p<0.01) and RV global strain (HR=1.16, 95% CI 1.04-1.30, p<0.01) were significantly associated with 30-day mortality. In multivariable Cox regression analysis, previous CHF (HR=17.9, 95% CI 2.65-120.7, p<0.01), GFR (HR=0.97, 95% CI 0.94-0.99, p<0.05) and RV global strain was independently associated with 30-day mortality (HR=1.14, 95% CI 1.00-1.29, p<0.05). Adding RV global strain to model which consisted previous CHF and GFR was associated with significantly improved predictive capacity. Finally, RV global strain parameter showed good predictive capacity for 30-day mortality in ROC curve estimation (AUC=0.71, 95% CI 0.54-0.88, p<0.05).

**Conclusion:** The RV global strain parameter is independent and strong predictor of 30-day mortality in patients with STEMI complicated by cardiogenic shock and its possible target of acute unloading.

## A-28 The effect of adding lactate acid concentration and LVEF to identify cardiogenic shock. Comparison of baseline characteristic from the DANSHOCK register, IABP-SHOCK II and CULPRIT-SHOCK trial

### Nanna Udesen^1^, Jacob Eifer Møller^1^, Matias Greve Lindholm^1^, Hans Eiskjær^1^, Christian Juhl Terkelsen^1^, Lisette Okkels Jensen^1^, Lene Holmvang^1^, Anders Junker^1^, Henrik Schmidt^1^, Kristian Wachtell^1^, Holger Thiele^2^, Thomas Engstrøm^1^, Christian Hassager^1^

#### ^1^Odense University Hospital, Odense, Denmark; ^2^University of Leipzig, Leipzig, Germany

##### **Correspondence:** Nanna Udesen

**Background:** The classic criteria to diagnose cardiogenic shock based on measurement of cardiac output and left ventricular filling pressure are rarely available in the acute clinical setting of acute myocardial infarct related cardiogenic shock (AMICS). Thus, shock diagnosis is based on blood pressure and objective signs of tissue hypoperfusion. This study compares characteristics of patients where a mandatory increase of lactate> 2.5mmol/l as sign of tissue hypoperfusion and LVEF <45% was used to diagnose AMICS with patients with more classically diagnosis of AMICS criteria based on clinical signs of tissue hypoperfusion

**Hypothesis:**

**Methods:** The baseline characteristics for patients screened for inclusion in the DANSHOCK trial (n=414) were compared to patients randomized in the IABP-SHOCK II (n=595) and CULPRIT SHOCK trial (n=686).

**Results:** All patients except 6.7% in the DANSHOCK registry received primary percutaneous intervention (PCI). The median age ranged from 68-70 and male gender was predominant in all studies. Comorbidities were evenly distributed across groups. However, despite higher systolic blood pressure threshold in the DANSHOCK screening population (100 mmHg), baseline systolic and diastolic blood pressure was lower in the DANSHOCK population. The lactate values were highest in the DANSHOCK population, where all had values above 2.5 mmol/l. LVEF was found lowest in the DANSHOCK registry which also had the lowest number of patients resuscitated after OHCA included although the condition was frequently present in all groups ranging from 39.8% to 53.6%. Also the proportion of patients treated with catecholamines, requiring mechanical ventilation and renal replacement therapy was highest in patients screened for DANSHOCK compared with IABP-SHOCK II and CULPRIT-SHOCK patients.

**Conclusion:** Mandatory LVEF < 45% and lactate > 2.5 mmol/l to diagnose cardiogenic shock identifies an AMICS population with more profound shock compared with criteria based on clinical signs of hypoperfusion.

## A-29 Rational and Design of the PROTECT KIDNEY Trial

### Ralf Westenfeld, Julian Wiora, Max Spieker, Christian Jung, Patrick Horn, Malte Kelm

#### University Hospital Dusseldorf, Department of Cardiology, Dusseldorf, Germany

##### **Correspondence:** Ralf Westenfeld

**Background:** Impella-protected PCI may be considered if patients display characteristics of the three items: 1. complex anatomy for PCI, 2. reduced LVEF and 3. relevant comorbidities. A drawback for dissemination of protected PCI relates to the fact that objective scores, when to initiate protected PCI do not exist. Contrast induced acute kidney injury (CI-AKI) represents a complication affecting short- and long-term survival. Only recently, retrospective analyses deciphered that Impella-protected PCI is associated with procurement of kidney function. The Mehran score allows valid calculation or the risk for CI-AKI including clinical variables als used for decision making for protected PCI into a metric scale.

**Aims of the trial**: 1. To establish causality of kidney protection by Impella support. 2. To decipher pathomechanisms of Impella-mediated procurement of kidney function. 3. To collect evidence that avoiding CI AKI translates into improved long-term outcome.

**STAGE 1:** Pivotal trial to determine safety and feasibility of Impella-protected PCI in a cohort of CI AKI high risk patients. Forty patients with a Mehran above 26% will be randomized 1:1 to standard versus Impella-assisted PCI. Primary endpoint will be serum creatinine two days after PCI. Secondary endpoints will include bleeding and access site complications. Moreover, effects of Impella-assisted PCI on pathways for salt and water handling, as well as kidney oxygenation will be detected by sequential BOLD MRI imaging.

**STAGE 2** will evaluate the efficacy of Impella-assisted PCI to reduce the incidence of CI AKI. According to sample size calculations (CIA AKI incidence: control group 30%; Impella-assisted group 10%, alpha 0.05, Power 0.8, dichotomous endpoint, independent groups - 124 patients) a total of 150 patients with a Mehran score above 26% will be randomized 1:1 to standard PCI versus Impella-assisted PCI. Primary endpoint will be incidence of CI AKI (AKIN level 1). Secondary endpoints will be serum creatinine, eGFR (creatinine, cystatin), tubular injury (NGAL serum levels), need for dialysis. Exploratory endpoints may include kidney function and survival at 90 days.

## A-30 Systematic literature review: LV unloading for infarct size reduction

### Satoshi Miyashita, Olympia Bikou, Kelly Yamada, Carlos Santos-Gallego, Kenneth Fish, Roger J. Hajjar, Kiyotake Ishikawa

#### Icahn School of Medicine, New York, NY, USA

##### **Correspondence:** Satoshi Miyashita

**Background:** Recent emergence of percutaneous LVADs has made acute LV unloading a realistic approach to treat patients presenting with MI. The concept of LVAD use for infarct size reduction is not new and first study examining this concept was published more than 40-years ago.

**Hypothesis:** Literature review of previous LV unloading studies in MI will provide information on key factors for successful infarct size reduction.

**Methods:** A literature review was performed using PubMed to identify experimental studies of acute LVAD use (≤24 hours) in acute MI. Studies that reported infarct size or percent infarct size relative to area-at-risk were included.

**Results**: A total of 32 experimental animal studies (24 dog, 7 pig, and 1 sheep studies) were identified. Ischemia duration ranged from 15 minutes to 24 hours. Reperfusion was performed in 27 studies. All 5 studies without reperfusion found significant infarct size reduction with LVAD support. Overall, twenty-five studies reported significant infarct reduction compared to the control group (Positive studies), whereas other 7 studies reported no difference in infarct size (Negative studies). Positive studies showed a median of 53.3% (range 25.5-88.0%) relative reduction in infarct size against the control group. Type of species used, total ischemia time, total reperfusion time, size of the infarct size in the control groups were not different between the Positive and Negative studies. In the Positive studies, duration of ischemia time without LV support was significantly shorter (median: 1.5hr vs 2hr, P=0.005), LV support was initiated before reperfusion (19/21 vs 5/9, p=0.03), and LV assist involved blood withdrawal from the left heart (inlet placed in LA or LV or right heart inlet plus LV venting), (23/25 vs 2/5, P=0.004).

**Conclusion:** Analysis of pooled data in experimental animals strongly supports infarct size reduction effect of acute LV unloading. Factors important for maximizing this beneficial effect seems to be minimizing unsupported ischemia time, unloading prior to reperfusion, and sufficient volume reduction of the LV.

## A-31 Cardiac uptake and release of exosomes during altered LV load in ischemic HF

### Olympia Bikou, Shin Watanabe, Prabhu Mathiyalagan, Kelly Yamada, Divya Jha, Neha Agarwal, Satoshi Miyashita, Carlos Santos-Gallego, Roger J. Hajjar, Susmita Sahoo, Kiyotake Ishikawa

#### Icahn School of Medicine, New York, NY, USA

##### **Correspondence:** Olympia Bikou

**Background:** Intense research focusing on characterizing the hemodynamic and physiological impact of LV unloading has improved our understandings. However, humoral effects of acute unloading has not been well studied.

**Hypothesis:** Biological changes associated with unloading of the ischemic LV are partly mediated by local and remote communication via exosomal microRNAs.

**Methods:** Yorkshire swine were percutaneously induced anterior MI. One week after MI, pigs underwent 1 hour of LV unloading with Impella CP (n=3) or LV over-loading with percutaneous induction of aortic regurgitation (n=3). Blood was simultaneously withdrawn from the coronary artery (before cardiac circulation) and the coronary sinus (after cardiac circulation) before and after cardiac unloading/over-loading. Exosomes were isolated from the plasma using differential ultracentrifugation and microRNAs packaged in the exosomes are extracted. MicroRNAs were sequenced and transcardiac gradient was calculated by subtracting the mapped copy numbers.

**Results**: MicroRNAs with at least 5 counts for normalized CPM (counted per million) were included in the analysis. A total of 129 microRNAs after LV unloading and 220 microRNAs after LV over-loading were identified. Among them, 127 mircoRNAs were common in both. After LV unloading, there were 39 microRNAs that showed increase in cardiac uptake in all 3 pigs, whereas 7 microRNAs showed increase in cardiac release. After LV over-loading, there were 33 microRNAs that showed increase in cardiac uptake in all 3 pigs and 32 microRNAs that showed increase in cardiac release. Six microRNAs showed opposite transcardiac gradient after LV unloading and overloading (increased uptake after over-loading and increased release after unloading).

**Conclusion:** There are active uptake and release of exosomes in the ischemic heart. We identified several microRNAs that showed opposite response to LV unloading and over-loading, suggesting that these microRNAs are involved in load-dependent biological regulation of the cross-chamber and cross-organ communication.

## A-32 Impact of acutely increased load on post-MI heart

### Kelly Yamada, Olympia Bikou, Shin Watanabe, Prabhu Mathiyalagan, Kelly Yamada, Divya Jha, Neha Agarwal, Satoshi Miyashita, Carlos Santos-Gallego, Roger J. Hajjar, Susmita Sahoo, Kiyotake Ishikawa

#### Icahn School of Medicine, New York, NY, USA

##### **Correspondence:** Kelly Yamada

**Background:** Acute mechanical unloading offers beneficial effects on post-MI hearts. However, its mechanism and important factors remain to be fully characterized. To better understand biology, combination of gain- and loss-of-function studies are insightful. In view of “acute unloading” as a gain-of-function, loss-of-function shall be “acute over-loading”

**Hypothesis:** Cardiac physiological and molecular changes associated with acute over-loading offer improved characterization of cardiac load effects on post-MI heart.

**Methods:** Yorkshire pigs were percutaneously induced anterior MI. One week after MI induction, aortic regurgitation (AR) was percutanously induced to acutely over-load the heart. Pressure-volume data were evaluated before and after acute cardiac over-loading. Non-ischemic myocardial samples were collected after 3 hours of over-loading and markers of oxidative stress were compared to those from LV unloaded (Impella) animals.

**Results**: Severe AR was successfully induced in 6 pigs. One pig died from acute HF before LV pressure-volume assessment. Induction of AR significantly increased LV end-diastolic pressure (14.7±6.3 vs 27.0±4.8mmHg, P=0.03), end-diastolic volume (97.5±10.6 vs 106.3±10.1ml, P=0.02), and end-systolic volume (49.0±18.0 vs 60.1±13.7 ml, P=0.03). LV output remained unchanged despite severe AR (4.79±1.71 vs 4.63±0.78 L/min, P=0.74), while effective cardiac output (LV output- regurgitation flow) measured by the Swan-Ganz catheter was reduced (4.79±1.71 vs 3.32±0.7L/min, P=0.08). LVEF (53.0 ±15.0 vs 47.2±9.0 %, P=0.18), maximum dP/dt (2009±799 vs 1597±139mmHg/s, P=0.27) and LV stroke work (4288±1516 vs 3221±724ml*mmHg, P=0.06) tended to decline, whereas minimum dP/dt (-2041±524 vs 1202±162mmHg/s, P=0.009) and Tau (46.3±7.1 vs 57.7±10.7ms, P=0.02) showed significant deterioration. Markers of oxidative stress (8-hydroxy-deoxyguanosine and 3-nitrotyrosine) in the non-ischemic myocardium were increased after LV loading, whereas it was reduced in the LV that underwent unloading.

**Conclusion:** Post-MI heart was unable to compensate for decreased forward flow upon acute over-loading and both systolic and diastolic functions deteriorated. Non-ischemic myocardial tissue samples exhibited increased oxidative stress in contrast to reduced oxidative stress in the samples after LV unloading. These results support the importance of cardiac load in post-MI heart.

## A-33 Impella Is Associated With Reduced Incidence Of Contrast-Induced Nephropathy In Patients At Higher Risk For PCI

### Julian Wiora, Patrick Horn, Christian Jung, Susanne Wolters, Georg Wolff, Tobias Zeus, Malte Kelm, Ralf Westenfeld

#### University Hospital Dusseldorf, Department of Cardiology, Dusseldorf, Germany

##### **Correspondence:** Julian Wiora

**Background:** Demographics are shifting to an elderly population with more comorbidities potentially qualifying for mechanical circulatory support (MCS) during high-risk primary coronary intervention (PCI). In a previous work, a single center registry, we observed an association for lower incidence of contrast-induced nephropathy (CIN) in patients supported with Impella compared to vaECMO. Here, we performed a propensity score matching analysis comparing the Impella collective to a control group (CG) of patients in high-risk PCI setting without MCS to see, whether findings correlate to Impella-usage.

**Hypothesis:** Impella is superior towards a propensity matched non-Impella-control-group in high-risk PCI concerning patient’s safety as an association to lower incidence of contrast-induced nephropathy

**Methods:** All patients scheduled for elective high-risk PCI were included, retrospectively. Patients at higher risk were characterized by aortic stenosis, compromised ejection fraction, complex anatomy, etc. All patients turned down for surgery. Propensity score matching was performed for Impella-patients (n=35) in a 1-to-1-ratio CG (n=35) using Mehran Risk Score for predicting AKI and baseline GFR as determining factors. We analyzed PCI success; peripheral complications (bleeding, AV-fistula, aneurysma spurium) and CIN (defined as AKIN I or higher).

**Results:** Focus of post-hoc analysis lied on Mehran-Score, which did not differ with respect to predicted CIN [%] (CG 22.2±14.2 vs Impella 26.8±16.8; p=0.23) and GFR (59±20 ml/min, vs 68±23; p=0.09). Impella PCI were associated with a reduced incidence of CIN [%] (31 vs 9; p=0.02)

Patient characteristics did not differ with respect to comorbidities like arterial hypertension [%] (CG 94 vs Impella 89; p=0.39), diabetes mellitus [%] (34 vs 43; p=0.46), PAD [%] (66 vs 11, p=0.39), contrast media exposure [ml] (226±117 vs 262±88 mL; p=0.15). Control group was older [y] (79±9 vs 74±8; p=0.02) and had less compromised LV-F [%] (5±9 vs 45±14; p=0.03). PCI was more complex and challenging in Impella-cohort resulting in a larger amount of DES implanted (1.6±1.1 vs 2.5±1.1; p=0.001), longer procedure times, higher dosages and radiation times. Peripheral vascular complications did not reach statistical significance in advantage towards CG (1 vs 4; p=0.16).

**Conclusion:** It seems our earlier conclusion of a lower incidence of CIN in MCS-assisted high-risk PCI with Impella is valid in comparison to a propensity matched CG of patients in high-risk PCI setting without MCS.

## A-34 The impact of Impella LV unloading on invasive measures of myocardial supply and demand in patients undergoing high-risk percutaneous coronary intervention

### Natalia Briceno, Matthew Ryan, Kevin O’Gallagher, Howard Ellis, Tiffany Patterson, Bhavik Modi, Haseeb Rahman, Ian Webb, Brian Clapp, Antonis Pavlidis, Simon Redwood, Ajay Shah, Divaka Perera

#### St Thomas’ Hospital/King’s College London, London, UK

##### **Correspondence:** Natalia Briceno

**Background:** The Impella device is being increasingly used to support high-risk PCI. Whilst the differential effects of Impella on myocardial supply and demand have been described in animal and mathematical models, whether this translates to the clinical setting is incompletely understood.

**Hypothesis:** We hypothesized that Impella support would result in a greater impact on myocardial demand than supply, with maximal support resulting in reductions in LV stroke work and pressure volume area (PVA), with an overall significant beneficial effect on the supply:demand ratio.

**Methods:** 11 patients with severe ischaemic cardiomyopathy undergoing elective PCI with Impella (2.5 or CP) were recruited. Simultaneous target vessel intra-coronary pressure and Doppler flow velocity data and LV pressure volume loops were obtained following PCI, during Impella maximal support (P8) and minimal support (P1). Wave intensity analysis was performed to quantify the dominant diastolic microvascular-originating backward expansion wave (BEW). PVA, a measure of myocardial oxygen demand, was calculated as the sum of potential energy and LV stroke work. Myocardial supply:demand ratio was defined as the ratio between coronary flow and PVA.

**Results:** Impella support resulted in an increase in distal coronary pressure (81.2±16.9 vs. 89.8±17.5mmHg,p=0.005) with no effect on flow velocity (31.6±16.4 vs. 33.6±13.7 cm s-1, p=0.2). There was no difference in the magnitude of the peak BEW (p=0.91) with support. The aortic pulse pressure correlated with total wave area during maximal support, with a smaller pulse pressure resulting in lower total wave area (p=0.03, r2=0.43). There was a reduction in LV end diastolic pressure (p=0.04), stroke work (p=0.04) and PVA (p=0.03) with Impella maximal support, with a corresponding increase in the myocardial supply:demand ratio (3.7±1.5 x 102 vs. 4.7±1.5 x 102 mmHg-1.s-1.cm-2, p=0.01).

**Conclusion:** Impella support favourably impacts the myocardial supply: demand ratio during high-risk PCI, primarily via LV unloading rather than by improving coronary perfusion.

## A-35 Comparative effects of a smaller and larger-capacity intra-aortic balloon pump on coronary hemodynamics

### Natalia Briceno, Kalpa De Silva, Matthew Lumley, Howard Ellis, Matthew Ryan, Bhavik Modi, Balrik Kailey, Brian Clapp, Michael Marber, Simon Redwood, Divaka Perera

#### St Thomas’ Hospital/King’s College London, London, UK

##### **Correspondence:** Natalia Briceno

**Background:** Randomized data suggests a disconnect between physiological observations and clinical outcomes with routine use of a smaller capacity (40cc) IABP. A larger (50cc) capacity device has now been introduced, for presumed superior haemodynamic effect, but the comparative aortic and coronary effects are currently unknown.

**Hypothesis:** We hypothesized that the 50cc IABP would have a greater impact on coronary and aortic hemodynamics, with augmentation in coronary flow regardless of the autoregulatory state, driven by a larger late diastolic IABP-forward compression wave.

**Methods:** Ten patients with ischaemic cardiomyopathy (LVEF 28 ± 10%) were studied. Simultaneous intra-coronary pressure and Doppler measurements were undertaken after PCI, during unassisted and IABP-assisted conditions, first with the 40cc and then the 50cc balloon. Autoregulation was modulated by using intracoronary Adenosine. Coronary wave intensity analysis was performed to characterise and compare the wave energies associated with balloon counterpulsation

**Results:** There was no difference in the amount of aortic diastolic pressure augmentation (47.7 (43.4,62.0) vs. 63.1 (50.7,71.0) mmHg, p=0.30) between the 40cc and 50cc balloons respectively, with a numerical increase in systolic unloading seen with the 50cc balloon (p=0.08). During basal conditions, coronary flow was unchanged during 40cc IABC, but increased with the 50cc device (p=0.01), with a linear correlation with the deflation pressure (R2=0.81, p=0.0004). Both balloons increased coronary flow during maximal hyperemia. The late diastolic IABP-forward compression wave temporally associated with balloon inflation was numerically larger during 50cc counterpulsation (0.92 (0.73, 1.42) vs. 1.32 (0.83, 3.62) W m-2s-2 x105, p=0.06) and correlated with the degree of diastolic augmentation (R2=0.40, p=0.003).

**Conclusion:** The 50cc IABP has a more pronounced effect on aortic and coronary hemodynamics. Compared to the 40cc device, the 50cc balloon augments coronary flow in the presence of both intact and exhausted autoregulatory systems, which may allow it to provide a more consistent hemodynamic effect.

## A-36 Intra-aortic balloon counterpulsation for high-risk percutaneous coronary intervention: defining coronary responders

### Natalia Briceno, Kalpa De Silva, Matthew Ryan, Tiffany Patterson, Kevin O’Gallagher, Howard Ellis, Simone Rivolo, Jack Lee DPhil, Simon Redwood, Ajay M Shah, Michael Marber, Divaka Perera

#### St Thomas’ Hospital/King’s College London, London, UK

##### **Correspondence:** Natalia Briceno

**Background:** The effect of intra-aortic balloon counterpulsation (IABC) varies and it is unknown whether this is due to a heterogeneous coronary physiological response. This study aimed to characterise the coronary and left ventricular (LV) effects of IABC and define responders in terms of their invasive physiology.

**Hypothesis:** We hypothesized that a greater coronary response to IABC will be seen in those patients with more deranged systemic hemodynamics and greater ischaemic burden.

**Methods:** 27 patients (LVEF 31± 9%) scheduled for PCI with IABP support underwent coronary pressure and Doppler flow measurements in the target vessel and acquisition of LV pressure volume loops after PCI, with and without IABC. Coronary wave intensity analysis was performed to identify accelerating and decelerating waves and Perfusion Efficiency (PE) calculated as the proportion of total wave energy comprised of accelerating waves. Responders were defined as those with an increase in PE with IABC. The myocardial supply:demand ratio was defined as the ratio between coronary flow and LV pressure volume area (PVA).

**Results:** Percentage change in PE ranged from -32% to 100.3%, with 44.4% defined as responders. Responders were more likely to have undergone complex PCI (p=0.03) with a higher pre PCI disease burden (BCIS jeopardy score: 9.1 ± 2.5 vs. 11 ± 1.3, p=0.02) and had lower unassisted mean arterial (87.4 ± 11.0 vs. 77.8 ± 11.6, mmHg, p=0.04) and distal coronary pressures (88.0 ± 11.0 vs. 71.6 ± 12.4 mmHg, p<0.001). The effect of counterpulsation on stroke work and PVA was similar between responders and non-responders, with overall no effect of IABC seen on the myocardial supply and demand ratio (p=0.34)

**Conclusion:** IABC has minimal effect on demand but there is marked heterogeneity in the coronary response to IABC, with the greatest response observed in those patients with the most disordered autoregulation.

## A-37 Increasing incidence and complexity of cardiogenic shock after acute myocardial infarction related to change in patient population: a Danish retrospective cohort study

### Ole K. Moller-Helgestad, Jakob Josiassen, Christian Hassager, Lisette O. Jensen, Lene Holmvang, Anne Sørensen, Martin Frydland, Annmarie T. Lassen, Nanna L. J. Udesen, Henrik Schmidt, Hanne B. Ravn, Jacob E. Møller

#### Odense University Hospital, Odense, Denmark

##### **Correspondence:** Ole K. Moller-Helgestad

**Background:** To describe changes in annual incidence and patient population including management and short-term outcome of cardiogenic shock (CS) following acute myocardial infarction (MI) in Southeastern Denmark from 2010 to 2017.

**Hypothesis:**

**Methods:** Consecutive patients with acute MI and CS were identified retrospectively through Danish registries, including individual evaluation of medical records on all patients.

**Results:** A total of 1.716 CS patients were identified and annual incidence increased in the adult population (from a nadir 65.3 cases per million person-years in 2013 to 80.0 cases per million person-years in 2017, p for trend<0.001) driven by increase in patients initially surviving out of hospital cardiac arrest. Fewer patients revascularised for ST elevation MI developed CS (from 10.0% in 2010 to 6.6% in 2017, p for trend<0.001) and CS was present at an earlier stage with fewer developing CS >12 hours after admission over the study years (14.8% in 2010 to 6.9% in 2017, p for trend=0.04). Mean systolic blood pressure was lower (63 ± 11 mmHg in 2010 to 61 ± 13 mmHg in 2017, p for trend=0.001) and the frequency of a LVEF≤30% increased in recent years (61.8% in 2010 to 71.4% in 2017, p for trend=0.004). Use of intra-aortic balloon pump was almost completely abandoned in recent study years with a simultaneous increase in the use of Impella devices and veno-arterial extracorporeal membrane oxygenation. Annual thirty-day mortality for the entire CS cohort remained unchanged exceeding 50%.

**Conclusion:** Incidence of CS increased in the population and fewer with STEMI developed CS. Complexity and severity of shock increased during the study period with earlier presentation and more compromised haemodynamics. Overall survival remained unchanged.

## A-38 Left Ventricular Load is a Major Determinant of Myocardial Mitochondrial Structure and Function in Acute Myocardial Infarction

### Lija Swain, Xiaoying Qiao, Lara Reyelt, Shiva Annamalai, Natalia Briceno, Paige Crowley, Corinna Bealle, Aditya Chenjorwalla, Navin Kapur

#### Tufts Medical Center, Boston, MA, USA

##### **Correspondence:** Lija Swain

**Background:** Myocardial infarction (MI) leading to heart failure (HF) is a major cause of mortality worldwide. First reducing left ventricular workload using a TV-pump and delaying reperfusion (Primary Unloading) reduces myocardial infarct size by 40-50% in preclinical models. Mitochondrial function is a critical determinant of myocyte survival during acute MI and further post-MI HF is accompanied by reduced mitochondrial oxidative metabolism and a compensatory increase in glycolysis leading to a stressed metabolic phenotype.

**Hypothesis:** We hypothesized that left ventricular load is a major determinant of mitochondrial functional capacity in AMI.

**Results:** We first studied global gene expression within the infarct zone using a whole genome transcriptome approach and identified several molecular targets promoting cardioprotective signaling, cellular respiration, mitochondrial structural integrity and mitochondrial function in the Primary unloading group compared to the Primary Reperfusion. Next, we identified that expression of cardioprotective genes and genes associated with mitochondrial function and structure including NDUFA8, Cox7c, ATP5GL and UQCRB were preserved within the infarct zone after Primary Unloading, not Primary Reperfusion. To test the impact of LV load on mitochondrial function, adult male swine were subjected to left anterior descending artery occlusion for 90 minutes followed by either immediate reperfusion (Primary Reperfusion); LV unloading for 30 minutes with an Impella CP device, then reperfusion (Primary Unloading); or LV loading for 30 minutes with VA-ECMO then reperfusion (Primary Loading). Using the Agilent Seahorse Platform, we isolated mitochondria from the infarct zone of all animals and quantified oxygen consumption in response to various agonist and antagonists of electron transport chain (ETC) complexes. We identified that compared to Primary Reperfusion (IR) or Primary Loading, Primary Unloading significantly improved function of Complex 1 in the ETC (Figure 1).

**Conclusion:** We report for the first time that a Primary Unloading strategy may preserve mitochondrial structure and function in acute MI. An analysis of these functional pathways may identify novel molecular targets that may either obviate the need for or work synergistically with a mechanical heart pump to reduce infarct size and limit the onset of heart failure after a heart attack.

**Fig. 1 (abstract A-38).**
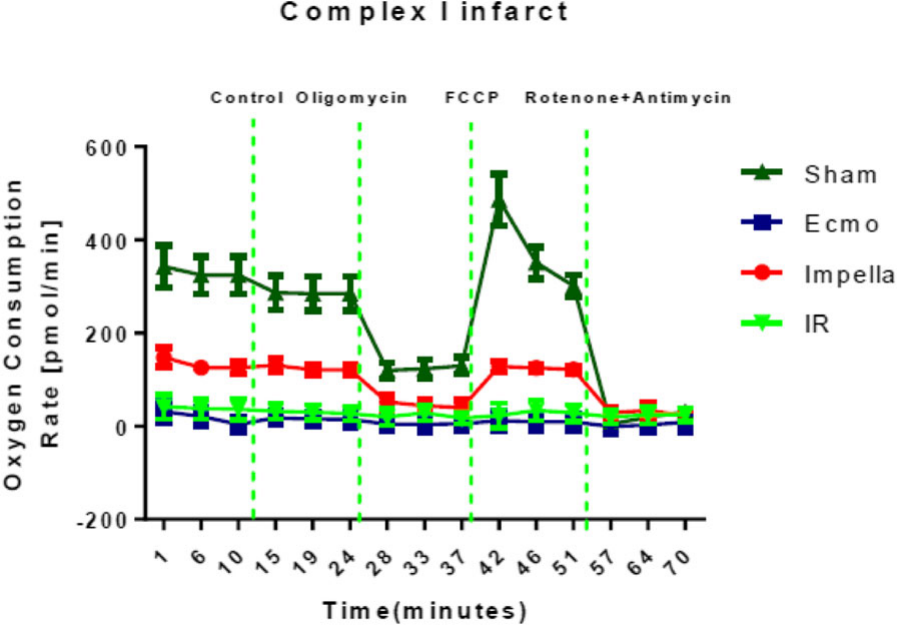
See text for description.

## A-39 Temporal trends, patterns, and long-term outcomes of post-myocardial infarction heart failure

### Ajar Kochar, Li Liang, Jacob A Doll, Jerry Curran, Eric D Peterson

#### Duke University Medical Center/Duke Clinical Research Institute, Durham, NC, USA

##### **Correspondence:** Ajar Kochar

**Background** Myocardial infarction (MI) is associated with several complications including heart failure (HF); there are limited data describing the contemporary prevalence of post-MI HF and long-term outcomes after post-MI HF.

**Hypothesis:** In the contemporary era, the rate of post-MI HF is increasing and it is associated with adverse 5 year outcomes.

**Methods:** We evaluated MI patients (defined as a primary or secondary ICD-9 code of 410.x1) aged ≥65 years between 2000 – 2013 in the Medicare claims database. Post-MI HF was defined as HF during index MI hospitalization or a hospitalization for heart failure within 1 year post-index MI admission (HF ICD-9 codes: 428.x, 402.x1, 404.x1, 404.x3). We evaluated 5-year mortality rates stratified by these two definitions of post-MI HF.

**Results:** The study population included 1,531,628 patients from 5,948 hospitals. The mean age was 78 years, 64.6% had NSTEMI and 35.4% experienced STEMI. Among these patients, 32.3% had HF during index admission and 10.4% had at least 1 hospitalization for HF within 1 year. The median time to first HF hospitalization was 66 days (Q1-Q3: 19-170 days). The temporal trend from 2000 – 2012 suggested mild reductions in the incidence of HF during index hospitalization (34.7% to 31.2%), HF hospitalization within 1 year (11.3% to 8.7%), and 1-year mortality (22.5% to 18.8%). Five-year mortality was: 79.7% for patients with HF at index admission and at least one HF hospitalization within 1 year, 68.2% for patients with a HF hospitalization within 1 year only, 66.2% for patients with HF during index admission only, and 38.4% without any heart failure.

**Conclusion:** Post-MI heart failure in older adults is common, occurring in 1 in 3 patients. Heart failure portends a significantly higher long-term mortality rate. Clinicians should aim to reduce the prevalence and severity of post-MI heart failure.

## A-40 Primary Left Ventricular Unloading Enhances Collateral Blood Flow and Reduces Infarct Size: A Preclinical Proof of Concept Study

### Shiva Annamalai, Natalia Briceno, Lara Reyelt, Lija Swain, Xiaoying Qiao, Robert Pedicini, Lena Jorde, Gemini Yesodharan, Corinna Beale, Divaka Perera, Navin Kapur

#### Tufts Medical Center, Boston, MA, USA

##### **Correspondence:** Shiva Annamalai

**Background:** The Collateral Flow Index (CFI) measures microcirculatory flow in myocardium subtended by an occluded coronary artery. The impact of left ventricular (LV) load on the CFI is unknown. The Impella pump reduces LV load, while veno-arterial extracorporeal membrane oxygenation (VA-ECMO) increases LV load.

**Hypothesis:** We hypothesized that LV unloading reduces myocardial infarct size, in part, by increasing the CFI in acute myocardial infarction (AMI).

**Methods:** The left anterior descending artery (LAD) was occluded via angioplasty for 90 min in adult Yorkshire swine (n=4-6/group). After 90 min of ischemia, the LAD was reperfused for 180 min in the primary reperfusion (PR) group. In the primary unloading (PU) and primary loading (PL) groups, an Impella CP or VA-ECMO was activated and the LAD left occluded for an additional 30 min, followed by 180 min of reperfusion. Using a pressure wire, the CFI was calculated during LAD occlusion as (Pw – RA)/(Pa – RA), where Pa, RA, Pw are aortic, right atrial and coronary wedge pressure. Myocardial infarct size was quantified using TTC.

**Results:** Following 90 min of LAD occlusion, there was no difference in CFI among the groups. After 30 minutes of PU, the CFI increased compared to pre-activation (+0.07 ± 0.03, p = 0.03) and compared to PR (-0.008 ± 0.02, p = 0.003) or PL (-0.02 ± 0.03, p = 0.001). PU reduced LV stroke work (LVSW) at 120 min compared to pre-activation (-26%, p=0.03) and compared to PR (-24%, p=0.03) and PL (-25%,p=0.03). Among all groups, the change in CFI between 90 and 120 min correlated inversely with the change in LVSW (r2 = 0.43, p = 0.01). PU reduced infarct size relative to the area at risk (35% ± 6%) compared to PR (51% ± 14%, p = 0.04) and PL (66% ± 13%, p = 0.0009). The change in CFI correlated inversely with infarct size (r2 = 0.42, p = 0.02).

**Conclusion:** Primary Unloading increases microcirculatory flow to ischemic myocardium and reduces infarct size. Compared to Impella unloading, VA-ECMO failed to increase the CFI and increased myocardial infarct size.

## A-41 VA-ECMO Increases Urinary Levels of the Biomarker Kidney Injury Marker-1 (KIM-1) in a Preclinical Model of Acute Myocardial Infarction

### Xiaoying Qiao, Lija Swain, Lara Reyelt, Cody Machen, Aditya Chennjorwala, Paige Crowley, Shiva Annamalai, Sina Foroutanjazi, Allen Razavi, Navin K Kapur

#### Tufts Medical Center, Boston, MA, USA

##### **Correspondence:** Xiaoying Qiao

**Background:** Irrespective of the underlying cause, acute kidney injury (AKI) is associated with increased morbidity and mortality. Use of Impella trans-valvular pumps and veno-arterial extracorporeal membrane oxygenation (VA-ECMO) for acute myocardial infarction (AMI) and cardiogenic shock is growing. The Impella transfers rotational kinetic energy to blood and generate flow from the left ventricle into the ascending aorta (LV unloading). VA-ECMO drains blood from the venous system and returns oxygenated blood into the descending aorta, thereby increasing LV afterload (LV Loading). The impact of these support strategies on renal blood flow and function remains poorly understood. We hypothesized that compared to the Impella pump, VA-ECMO is associated with increased renal injury in AMI.

**Hypothesis: none stated**

**Methods:** Adult male swine were subjected to left anterior descending artery occlusion for 90 minutes followed by either immediate reperfusion (IRI), Impella or VA-ECMO starting 30 minutes before reperfusion, or sham-operated controls (n=4/group).

**Results:** Urinary levels of the biomarker kidney injury molecular 1 (KIM-1) were increased by VA-ECMO, not Impella (Figure). KIM-1 protein expression for precursor KIM-1 and the extracellular domain (soluble) of KIM-1 were analyzed by Western blot (Figure). Compared to sham, IRI and ECMO reduced levels of soluble KIM-1 and increased levels of the KIM-1 protein precursor in the renal cortex. Impella had no impact on KIM-1 protein levels.

**Conclusion:** This is first study to identify that VA-ECMO, not Impella, increases urinary levels of KIM-1, a highly sensitive biomarker of acute kidney injury, through a mechanism that may involve shedding of the KIM-1 extracellular domain form the renal cortex.

**Fig. 1 (abstract A-41).**
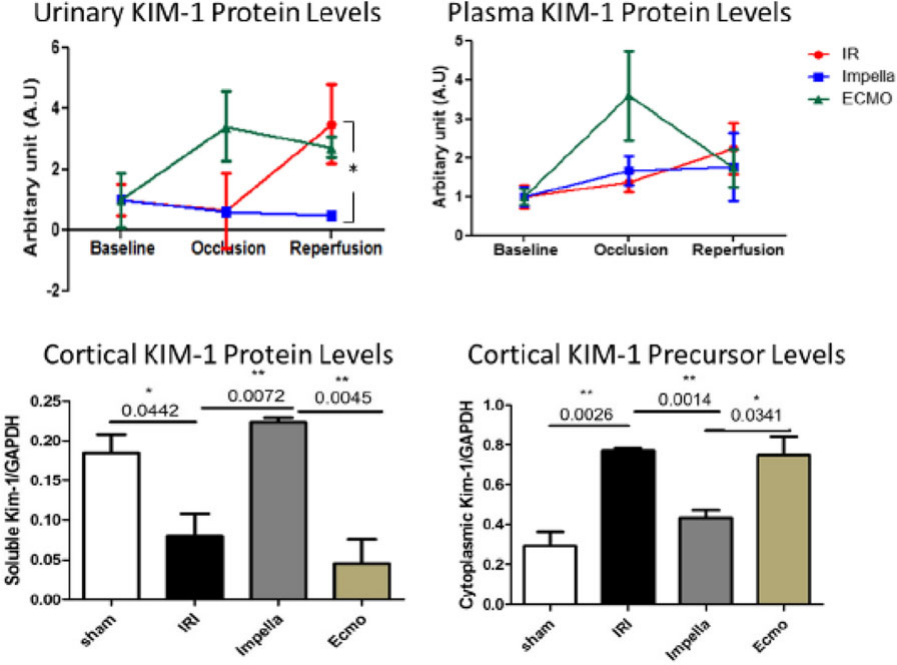
See text for description.

## A-42 Percutaneous Superior Vena Caval Occlusion for the Treatment of Acutely Decompensated Heart Failure

### Lara Reyelt^1^, Navin K. Kapur^1^, Shiva Annamalai^1^, Lena Jorde^1^, Rob Pedicini^1^, Peter Natov^1^, Carey Kimmelstiel^1^, Richard H. Karas^1^, Dan Burkhoff^2^

#### ^1^Tufts Medical Center, Boston, MA, USA; ^2^Columbia Medical Center, New York, NY, USA

##### **Correspondence:** Lara Reyelt

**Background:** Irrespective of cardiac output, elevated cardiac filling pressures is a major determinant of outcomes in acutely decompensated heart failure (ADHF). Current therapy for ADHF focuses on diuretic therapy, which may promote renal injury and delay optimization of cardiac filling pressures. We previously demonstrated that superior vena caval (SVC) occlusion is an effective mechanical method to reduce preload and increase cardiac output in a swine model of heart failure. We now explored the safety and feasibility of SVC occlusion in patients with acutely decompensated heart failure.

**Hypothesis:** We now explored the safety and feasibility of SVC occlusion in patients with acutely decompensated heart failure.

**Methods:** Patients with NYHA Class III-IV decompensated heart failure referred for right and left heart catherization were enrolled into 2 cohorts. Using a 30cc Coda endovascular balloon, Cohort A received SVC occlusion for 5 minutes only (n=4). Cohort B received intermittent SVC occlusion for 5 minutes and 10 minutes consecutively (n=1 of 3 completed). Hemodynamics were measured at baseline, at 1 minute intervals during occlusion, and 5 minutes after release of occlusion in both groups. An SVC venogram was performed after release of occlusion to assess for vessel integrity. Neurologic evaluation including Mini-Cog and NIH Stroke Scale (NIHSS) scores were performed before, during, and after SVC occlusion at 1, 4, and 24 hours.

**Results:** SVC occlusion was successfully performed in all patients. No neurologic side effects were noted. Mean arterial pressure (MAP) was unchanged throughout occlusion time. Right internal Jugular (IJ) venous pressure rose from 19 ± 1 to 44 ± 5 mmHg (p = 0.014) at 5 min and returned to baseline values after release of SVC occlusion. Compared to baseline, 5 minutes of SVC occlusion reduced right atrial pressure (RA; 22 ± 3 vs. 9 ± 1 mmHg, p = 0.003), pulmonary artery systolic pressure (PASP; 57 ± 13 vs. 36 ± 17 mmHg, p = 0.02), pulmonary artery diastolic pressure (PADP; 24 ± 13 vs. 14 ± 8 mmHg, p = 0.001), mean pulmonary artery pressure (mean PA; 36 ± 13 to 25 ± 14 mmHg, p = 0.014) and pulmonary capillary wedge pressure (PCWP; 32 ± 14 vs. 15 ± 8 mmHg, p = 0.001). Cardiac output after 5 min of SVC occlusion was non-significantly increased (4 ± 0.4 vs 5 ± 0.7, p=NS).

**Conclusion:** SVC occlusion is a novel approach to mechanically reduce cardiac filling pressures and pulmonary arterial pressure in the setting of acutely decompensated heart failure. We anticipate completion of Cohort B inclusive of both 5 and 10 min of SVC occlusion for final presentation. A novel system for SVC occlusion (Precardia, MD Start) is currently in development.

## A-43 Normal Serum Lactic Acid is Discordant with Shock in Advanced Heart Failure Patients

### Nikhil Narang, Ben Chung, Ann Nguyen, Imo Ebong, Luise Holzhauser, Imo Ebong, Jayant Raikhelkar, Nitasha Sarswat, Gene Kim, Sara Kalantari, Bryan Smith, Gabriel Sayer, Nir Uriel

#### University of Chicago, Chicago, IL, USA

##### **Correspondence:** Nikhil Narang

**Background**: Elevated lactic acid (LA) levels carry a poor prognosis in patients admitted with shock. Data is lacking on the association of LA level with the severity of decompensated heart failure (HF).

**Hypothesis:** This study assesses the relationship between LA levels, invasive hemodynamics and clinical outcomes.

**Methods:** Patients presenting to the cardiac care unit with decompensated HF between 2015-18 were prospectively enrolled into an invasive hemodynamics study. LA (normal 0.7-2.1 mmol/L) levels were obtained within 12 hours prior to right heart catheterization (RHC). No significant changes in therapy were made in the time between LA level collection & RHC. Patients were divided into 4 groups: (1, N=9) normal pulmonary capillary wedge pressure (PCWP) (< 18 mmHg)/ normal Fick cardiac index (CI) (≥ 2.2 L/min/m2), (2; N=3) normal PCWP/ low CI (< 2.2 L/min/m2), (3; N=20) elevated PCWP (≥ 18 mmHg )/ normal CI, (4; N=48) elevated PCWP / low CI.

**Results** 80 patients were enrolled. Mean age 58±14 years; 78% male, left ventricular ejection fraction was 24±4%. Prior to RHC, 55% patients were on vasoactives and/or inotropes. Mean (SD) PCWP was 26 ± 8.8 mmHg and mean CI was 2.06 ± 0.61 L/min/m2. 90-day all cause mortality was 33%. Overall, 81% had normal LA (≤2.1 mmol/L) prior to RHC. There was no correlation between LA level and PCWP (R=0.12; p=0.30); there was a moderate inverse correlation between LA level and CI (R=-0.40; p<0.01). Only 25% of patients with the highest risk hemodynamic profile (elevated PCWP/low CI) had an elevated LA level. When LA was stratified by tertile, there was no significant difference in mortality between the first and third tertile (p=0.28). However, mortality increased with worsening hemodynamics (elevated PCWP/ low CI; p=0.02).

**Conclusion:** In patients with decompensated HF, normal LA levels do not exclude the presence of cardiogenic shock with profoundly impaired cardiac output. Invasive assessment of hemodynamics should not be delayed based on LA level alone.

## A-44 Impact of Clinical Indication / Risk Strata on Outcomes in Patients Supported with Impella Microaxial Heart Pumps

### Elisha Wali, Anupama Joseph, Paul Larsen, Joseph Venturini, Taishi Hirai, Jonathan Rosenberg, John Blair, Jonathan Paul, Atman Shah, Sandeep Nathan

#### University of Chicago, Chicago, IL, USA

##### **Correspondence:** Elisha Wali

**Background:** As indications for mechanical circulatory support (MCS) with Impella have evolved, complications and mortality across indications remain incompletely understood.

**Hypothesis:** We hypothesized that mortality and complication rates would be significantly higher in patients with high-risk indications for Impella placement.

**Methods:** A retrospective study of pts treated with Impella over a 10-year period at a single large medical center was conducted. EMR were reviewed for pertinent clinical and procedural data.

**Results:** 127 consecutive patients with Impella were analyzed and divided into risk strata based on acuity of primary MCS indication: Group 1 - cardiac arrest (n=25), Group 2 - cardiogenic shock (n=41) or VT storm (n=2), Group 3 - ACS (n=20), Group 4 - high-risk PCI (n=34) or VT ablation (n=5). Impella 2.5L devices were utilized more frequently than Impella CP in Groups 3 (100% vs 0%) and 4 (72% vs 29%) (p<0.001). 30-day mortality was 72%, 58%, 32%, and 2.6% in groups 1-4 respectively (p<0.0001). Hemorrhage requiring transfusion occurred more frequently in higher acuity groups (32% in group 1, 23% in group 2, 15% in group 3, and 2.6% in group 4; p=0.006), with a trend toward more hemolysis (p=0.061) and limb ischemia (p=0.068) in higher acuity groups as well. In the total cohort, hemolysis (OR 6.8 [1.7-40.0]; p=0.003) and hemorrhage (OR 3.3 [1.2-9.9]; p=0.016) were univariate predictors of 30-day mortality, while limb ischemia (OR 3.2 (0.4-36.6); p=0.213) was not a significant predictor.

**Conclusion:** Patients with high acuity indications for Impella, had dramatically higher 30-day mortality and major complications. As indications for Impella use expand, future investigations should focus on optimization of risk and benefit in high-risk patient cohorts.

## A-45 Prognostic Value of Right Ventricular Function in Percutaneous Veno-Arterial Extracorporeal Membrane Oxygenation

### Anupama Joseph, Joseph Venturini, John EA Blair, Jonathan Paul, Atman Shah, Sandeep Nathan

#### University of Chicago, Chicago, IL, USA

##### **Correspondence:** Anupama Joseph

**Background:** Little is known about the impact of RV function on clinical outcomes in patients supported with percutaneous veno-arterial membrane oxygenation (pVA-ECMO). We characterized RV hemodynamic trends in pVA-ECMO patients and assessed the impact of specific parameters on clinical outcomes.

**Hypothesis:** Patients with favorable RV hemodynamics will have increased survival to discharge; similar bleeding, neurologic and limb complications; and will be more likely to successfully decannulate from pVA-ECMO.

**Methods:** We retrospectively identified patients receiving pVA-ECMO at our institution over a 10 year period and selected patients with complete hemodynamic, clinical, demographic and outcome data. Fisher’s exact test was used to analyze categorical variables and Student’s two-tailed t-test was used for continuous variables.

**Results:** Thirty patients undergoing pVA-ECMO cannulation primarily for LV shock, with simultaneous RV hemodynamic monitoring, were included. Patients successfully decannulated had a lower mean right atrial to pulmonary capillary wedge pressure ratio (RA:PCWP) at 48 (0.61 vs 0.91, p=0.01) and 72 hours (0.4 vs. 1.0, p=0.02) post cannulation, lower mean TPG (3.7 vs 7.2, p=0.02) and higher mean pulmonary artery pulsatility index (PAPi) at 72 hours (2.16 vs 1.31, p=0.04) post cannulation than those unable to be weaned. More patients survived to discharge with a higher mean PAPi at 72 hours (2.64 vs 1.41, p<0.001) and lower mean transpulmonary gradient (TPG) at time of implant (3.0 vs 12.5, p=0.03). Higher mean PAPi at 72 hrs trended toward decreased bleeding (1.96 vs 1.36, p=0.098).

**Conclusion:** Favorable RV hemodynamics may serve as a novel predictor of weaning success and increased survival to discharge in patients supported with pVA-ECMO. The impact of RV energetics in LV- versus BiV-failure requires further study.

## A-46 Cost-Utility of Extracorporeal Cardiopulmonary Resuscitation (ECPR) in Patients with Cardiac Arrest

### Murtaza Bharmal, Joseph Venturini, Rhys Chua, Willard Sharp, David Beiser, Corey Tabit, Taishi Hirai, Jonathan Rosenburg, Janet Friant, John Blair, Jonathan Paul, Sandeep Nathan, Atman Shah

#### University of Chicago, Chicago, IL, USA

##### **Correspondence:** Murtaza Bharmal

**Background:** Extracorporeal cardiopulmonary resuscitation (ECPR) provides hemodynamic and respiratory support and serves as a bridge to definitive therapy or to recovery. However, ECPR is resource-intensive and robust evidence of clear survival benefit is lacking. In this study, we investigated the cost-utility of ECPR (cost/QALY) in cardiac arrest patients treated at our institution.

**Hypothesis:** ECPR in patients with refractory cardiac arrest results in a meaningful quality-of-life as assessed by the standardized health-utilities-indices and is cost-effective.

**Methods:** We performed a retrospective review of ECPR patients who suffered cardiac arrest at our institution between 2012 to 2018. All medical care-associated charges with ECPR and subsequent hospital admission were recorded, including direct, indirect, total operating costs and payer charges. The quality-of-life of survivors was assessed with the Health Utilities Index Mark II. The cost-utility of ECPR was then calculated with cost and quality-of-life data.

**Results:** ECPR was used in 32 patients (15/32 in-hospital, 47%) with an average age of 52.5 16.3 years (59% male, 66% African American). The median duration of ECPR support and total length of stay was 2.1 days and 4.3 days, respectively. Survival to hospital discharge and 1-year survival were each 16%. The mean score of the Health Utilities Index Mark II at discharge for the survivors was 0.47 0.24 (range, 0.22-0.85). The average and median operating cost for patients undergoing ECMO were $156,263 and $125,683 per patient, respectively. The calculated cost-utility for ECPR was $65,088/QALY gained.

**Conclusion:** The calculated cost-utility for ECPR is within the threshold considered cost-effective in the United States (<$100,000/QALY gained). These results are comparable to the cost-effectiveness of heart transplantation for end-stage heart failure. Larger studies are needed to assess the cost-utility of ECPR and to identify whether other factors, such as patient characteristics and type of cannula, affect the cost-utility benefit.

## A-47 Outcomes Following Use of Percutaneous Mechanical Circulatory Support in Patients with In-Hospital Cardiac Arrest

### Sandeep Nathan, Eisha Wali, Anupama Joseph, Paul Larsen, Joseph Venturini, Taishi Hirai, Jonathan Rosenberg, John Blair, Jonathan Paul, Atman Shah, Sandeep Nathan

#### University of Chicago, Chicago, IL, USA

##### **Correspondence:** Sandeep Nathan

**Background:** The impact of percutaneous mechanical circulatory support (pMCS) with percutaneous veno-arterial extracorporeal membrane oxygenation (pVA-ECMO) and/or Impella on survival and complications in patients with in-hospital cardiac arrest (IHCA) is unclear.

**Hypothesis** We hypothesized that patient outcomes would vary based on type of pMCS.

**Methods:** A retrospective study of all IHCA patients treated with pMCS over a 10-year period was conducted at a single large medical center. Patients were included if clinical and procedural data were complete.

**Results:** In 49 patients with IHCA requiring pMCS, pVA-ECMO alone (n=31) was used more than Impella alone (n=13) or both combined (EC-pella, n=5). Patients with AMI (n=16) or status-post PCI (n=15) were more likely to receive Impella alone (p=0.001 and 0.006 respectively). Impella patients had more CAD (p=0.004); prevalence of CKD, HTN, DM, and CHF was similar. There was no difference in pre-IHCA SOFA scores (p=0.8). Dwell time was longer with EC-pella than Impella alone (6.6±5.9 vs 1.6±1.6 days; p=0.016). There was no significant difference in 30-day survival (p=0.7). Survival to discharge was 0% with EC-pella, 26% with pVA-ECMO, and 36% with Impella (p=0.345). EC-pella patients had significantly more bleeding requiring transfusion than Impella patients (p=0.025) and a trend for more limb ischemia (p=0.066). Major bleeding strongly predicted decreased survival to discharge (0% vs 35.1% survival; p=0.012).

**Conclusion:** In IHCA patients with similar pre-arrest SOFA scores, 30-day mortality and survival to discharge with VA-ECMO vs Impella vs EC-pella were not significantly different. Impella alone was used more often for IHCA due to AMI or requiring PCI and was associated with shorter dwell times and less hemorrhage than EC-pella.

## A-48 Hemodynamic Effects of Mechanical Circulatory Support Devices in Ventricular Septal Defect: simulation-based comparison of different devices

### Mohit Pahuja^1^, Benedict Schrage^1^, Dirk Westermann^1^, Daniel Burkhoff^2^

#### ^1^Detroit Medical Center, Detroit, MI, USA; ^2^Columbia Medical Center, New York, NY, USA

##### **Correspondence:** Mohit Pahuja

**Background:** Ventricular septal defect (VSD) is an infrequent but lethal complication of AMI cardiogenic shock (CS), with mortality reported between 40-80%. Current information about the use of different forms of percutaneous mechanical circulatory support (pMCS) come from case reports and small case series. However, reports comparing hemodynamic effects of different forms of pMCS have not been performed and the optimal approach is not known.

**Hypothesis:** We hypothesized that direct LV unloading with a percutaneous left ventricular assist device (pLVAD) will more effectively reduce left-to-right shunting and provide better unloading of the LV compared to other forms of pMCS, especially ECMO, which is known to increase LV afterload and therefore has the potential to increase right-to-left shunting.

**Methods:** A literature search provided baseline hemodynamic characteristics (e.g., pulmonary flow (Qp), systemic flow (Qs), Qp/Qs, MAP, CVP, PAP and PCWP) of patients presenting with AMI-shock complicated by VSD. Parameters values of a previously describe comprehensive cardiovascular simulation (Harvi) were set to match this average literature-derived hemodynamics. Different forms of pMCS were imposed and hemodynamic effects compared.

**Results:** The simulated hemodynamic effects of VSD for the “average” patient are summarized in the Table; the impact on RV and LV mechanics are detailed by pressure-volume (PV) analysis in Fig. 1. With a VSD, the LV PV loop tilts leftward and the RV PV loop tilts rightward, indicating ejection from LV to RV throughout systole. There were increases in LV and RV EDP and peak RVP, and decreases in peak LVP and MAP. The impact of different forms of pMCS on hemodynamics are summarized in Table 1. Fig. 2 compares the effects of ECMO and pLVAD on pressure volume loops.

**Conclusion:** In this simulation study, ECMO and pLVAD provided equivalent total blood flow to the periphery; ECMO increased VSD flow and PCWP whereas these were decreased by pLVAD. The combination of pLVAD and ECMO provided the greatest increase in flow to the body and blood pressure, but did not significantly change PCWP or flow through the shunt compared to pLVAD alone.

**Fig. 1 (abstract A-48).**
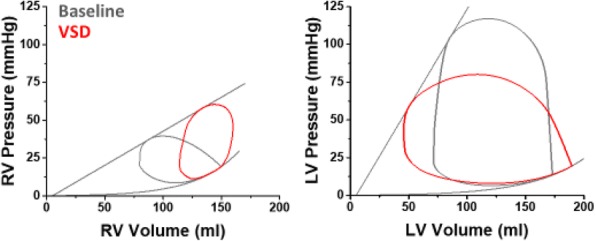
Simulated pressure-volume loops without (gray) and with (red) a ventricular septal defect.

**Table 1 (abstract A-48).** See text for description.

	Baseline	IABP	ECMO	LA-->Ao	pLVAD (5.0)	ECMO + pLVAD (5.0)
LV CO (L/min)	3.9	4.4	2.1	1.6	0.5	0.0
LVAD Flow (L/min)	n/a	n/a	3.5	3.5	4.9	3.4 + 4.6
VSD Flow (L/min)	6.6	5.8	8.9	6.5	5.2	6.2
PA Flow (L/min)	10.4	10.2	11.0	11.5	10.6	10.8
Total CO to Body (L/min)	3.9	4.4	5.6	5.1	5.5	8.0
Qp: Qs	2.7	2.3	2.0	2.3	1.9	1.4
CVP (mmHg)	17.0	17.0	16.0	18.0	18.0	17.0
PA mean (mmHg)	39.6	38.1	42.5	37.4	37.1	37.3
PCWP (mmHg)	24.9	23.7	26.9	21.1	22.1	22.0
MAP (mmHg)	62.2	68.5	79.1	76.1	78.9	106.0
Venous O2 Sat (%)	68.0	71.0	79.0	75.0	75.0	83.0

**Fig. 2 (abstract A-48).**
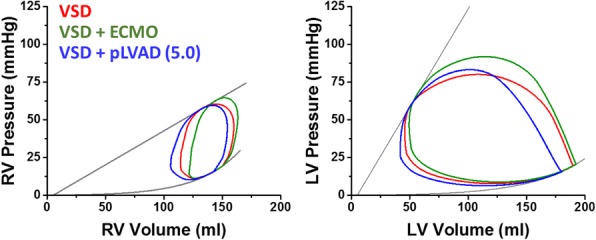
See text for description.

